# Synthesis and Biological Evaluation of Phenanthrenes as Cytotoxic Agents with Pharmacophore Modeling and ChemGPS-NP Prediction as Topo II Inhibitors

**DOI:** 10.1371/journal.pone.0037897

**Published:** 2012-05-29

**Authors:** Chia-Lin Lee, Ying-Ting Lin, Fang-Rong Chang, Guan-Yu Chen, Anders Backlund, Juan-Chang Yang, Shu-Li Chen, Yang-Chang Wu

**Affiliations:** 1 School of Chinese Medicine, China Medical University, Taichung, Taiwan; 2 Natural Medicinal Products Research Center, China Medical University Hospital, Taichung, Taiwan; 3 Department of Biotechnology, Kaohsiung Medical University, Kaohsiung, Taiwan; 4 Graduate Institute of Natural Products, Kaohsiung Medical University, Kaohsiung, Taiwan; 5 Cancer Center, Kaohsiung Medical University Hospital, Kaohsiung, Taiwan; 6 Division of Pharmacognosy, Department of Medicinal Chemistry, BMC, Uppsala University, Uppsala, Sweden; 7 Center for Molecular Medicine, China Medical University Hospital, Taichung, Taiwan; Univ of Bradford, United Kingdom

## Abstract

In a structure-activity relationship (SAR) study, 3-methoxy-1,4-phenanthrenequinones, calanquinone A (**6a**), denbinobin (**6b**), 5-OAc-calanquinone A (**7a**) and 5-OAc-denbinobin (**7b**), have significantly promising cytotoxicity against various human cancer cell lines (IC_50_ 0.08–1.66 µg/mL). Moreover, we also established a superior pharmacophore model for cytotoxicity (*r* = 0.931) containing three hydrogen bond acceptors (HBA1, HBA2 and HBA3) and one hydrophobic feature (HYD) against MCF-7 breast cancer cell line. The pharmacophore model indicates that HBA3 is an essential feature for the oxygen atom of 5-OH in **6a–b** and for the carbonyl group of 5-OCOCH_3_ in **7a–b**, important for their cytotoxic properties. The SAR for moderately active **5a–b** (5-OCH_3_), and highly active **6a–b** and **7a–b**, are also elaborated in a spatial aspect model. Further rational design and synthesis of new cytotoxic phenanthrene analogs can be implemented via this model. Additionally, employing a ChemGPS-NP based model for cytotoxicity mode of action (MOA) provides support for a preliminary classification of compounds **6a–b** as topoisomerase II inhibitors.

## Introduction

**Figure 1:**
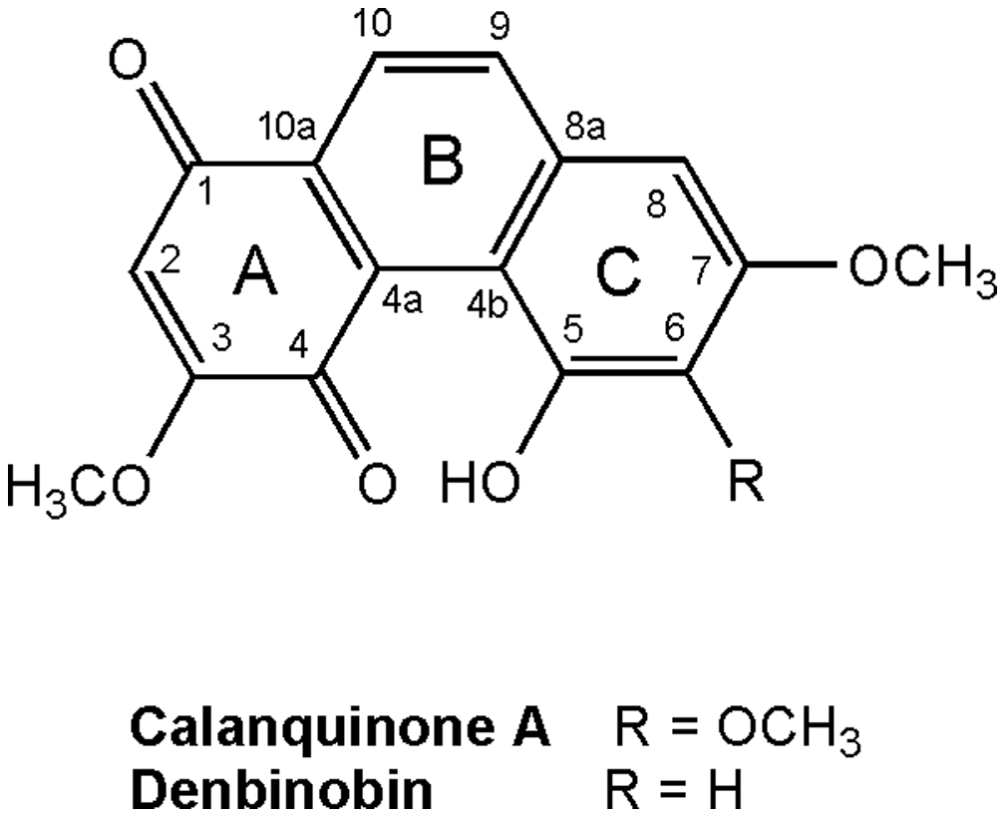
Structures of calanquinone A and denbinobin.

Natural phenanthrenes are probably generated from photochemical cyclization of stilbenes [1]. More than 270 phenanthrenes have been isolated from natural products, especially the family Orchidaceae, and some of them possess various biological activities, including cytotoxicity, antiplatelet aggregation, anti-inflammatory, antimicrobial, spasmolytic, anti-allergic activities and phytotoxicity [1]. In our previous study, calanquinone A [5-hydroxy-3,6,7-trimethoxy-1,4-phenanthrenequinone (5-hydroxy-3,6,7-trimethoxy-1,4-PQ)] (Figure 1), a new PQ isolated from *Calanthe arisanensis* in 2008, showed significant cytotoxic activity against human lung (A549), prostate (PC-3 and DU145), colon (HCT-8), breast (MCF-7), nasopharyngeal (KB), and vincristine-resistant nasopharyngeal (KBVIN) cancer cell lines with EC_50_ values of 0.03–0.45 µg/mL [2], [3]. Calanquinone A is related in structure to other naturally occurring cytotoxic PQs, including denbinobin (Figure 1) (5-hydroxy-3,7-dimethoxy-1,4-PQ), sphenone A (3,6,7-trimethoxy-1,4-PQ), densiflorol B (7-hydroxy-2-methoxy-1,4-PQ), and annoquinone A (3-methoxy-1,4-PQ) [1]. Denbinobin, first isolated from *Dendrobium nobile* in 1981, is the only 1,4-PQ that has been studied in terms of the cytotoxic mechanisms of human colon (HCT-116 and COLO 205), lung adenocarcinoma (A549), myelogenous leukemia (K562), and pancreatic adenocarcinoma (BxPC-3) cancer cell lines [4]–[11]. The data implied that denbinobin could be a potential anticancer lead compound.

In our preliminary results of cytotoxic structure-activity relationship (SAR) studies, calanquinone A (**6a**) displayed an up to 7-fold greater cytotoxic activity than denbinobin (**6b**), which is known as a potent cytotoxic agent [4]–[11], toward human liver (HepG2 and Hep3B), oral (Ca9-22), lung (A549) and breast (MEA-MB-231 and MCF7) cancer cell lines. Up to now, the SAR of PQs and phenanthrenes has only rarely been reported and is worthy of further study. In this research, calanquinone A (**6a**), denbinobin (**6b**) and their derivatives were synthesized [3], [12], [13] and evaluated for cytotoxic activity. In addition, employing a ChemGPS-NP based model provides the prediction for cytotoxicity mode of action (MOA) of calanquinone A (**6a**) and denbinobin (**6b**).

## Results and Discussion

### Chemistry

**Figure 2:**
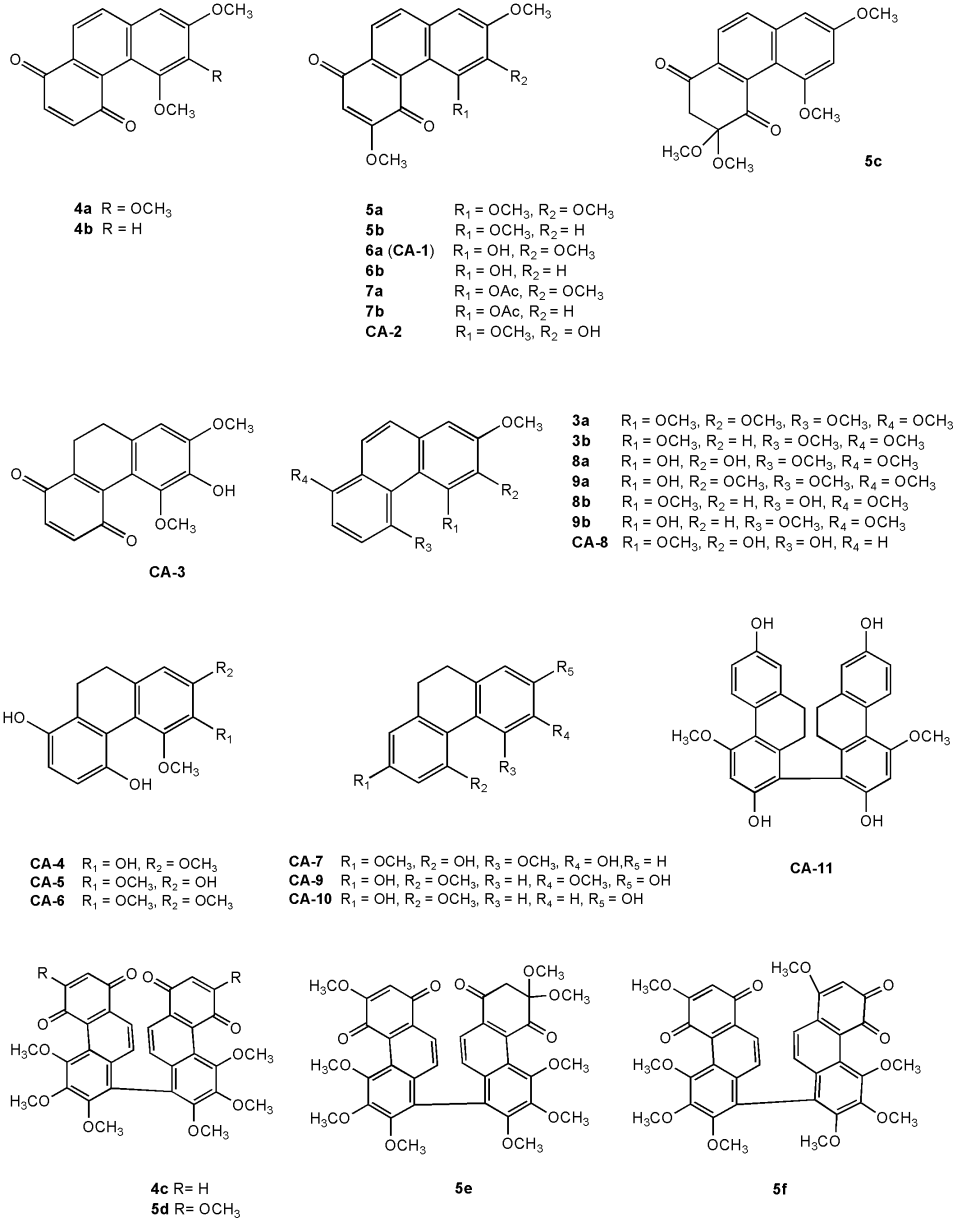
Structural sets used in the pharmacophore study. CA-1∼CA-11 and 3a-9b are noted as natural and synthesized compounds, respectively.

Eleven natural phenanthrene analogs (**CA-1**-**11**) (Figure 2) were isolated from *C. arisanensis*, and calanquinone A (**CA-1**) exhibited the highest potency against human cancer cell lines [2], [3]. According to the previous results [3], calanquinone A (**CA-1**) was selected as a lead compound and its derivatives were then synthesized for this study.

**Figure 3:**
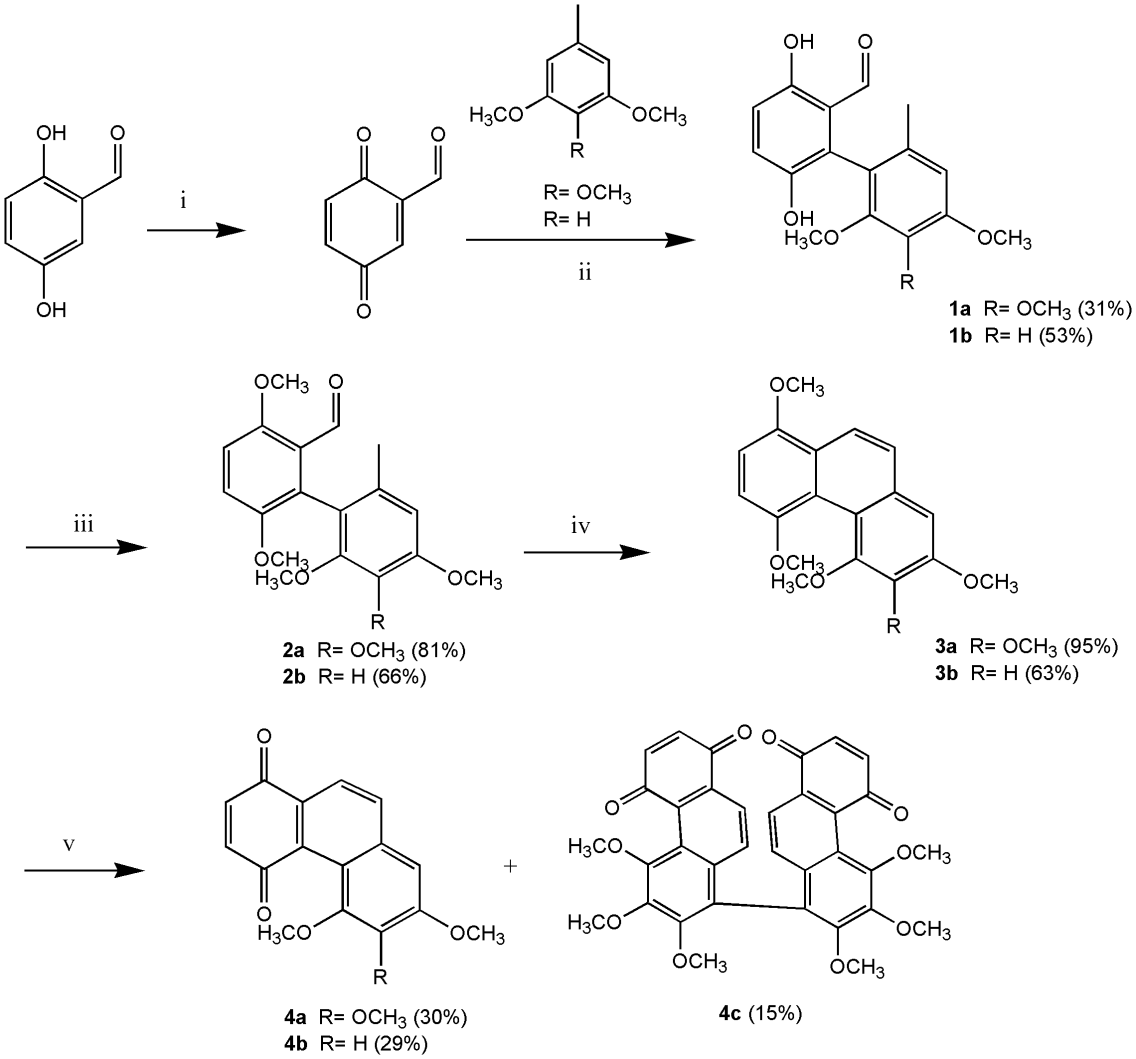
Synthetic procedure of phenanthrene derivatives. Reagents and conditions: (i) DDQ, benzene, RT. (ii) TFA, ether, RT. (iii) Me_2_SO_4_, K_2_CO_3_, acetone, reflux. (iv) P_4_-*t*Bu, benzene, 140°C. (v) AgO, 6 N HNO_3_, acetone, 60°C.

**Figure 4:**
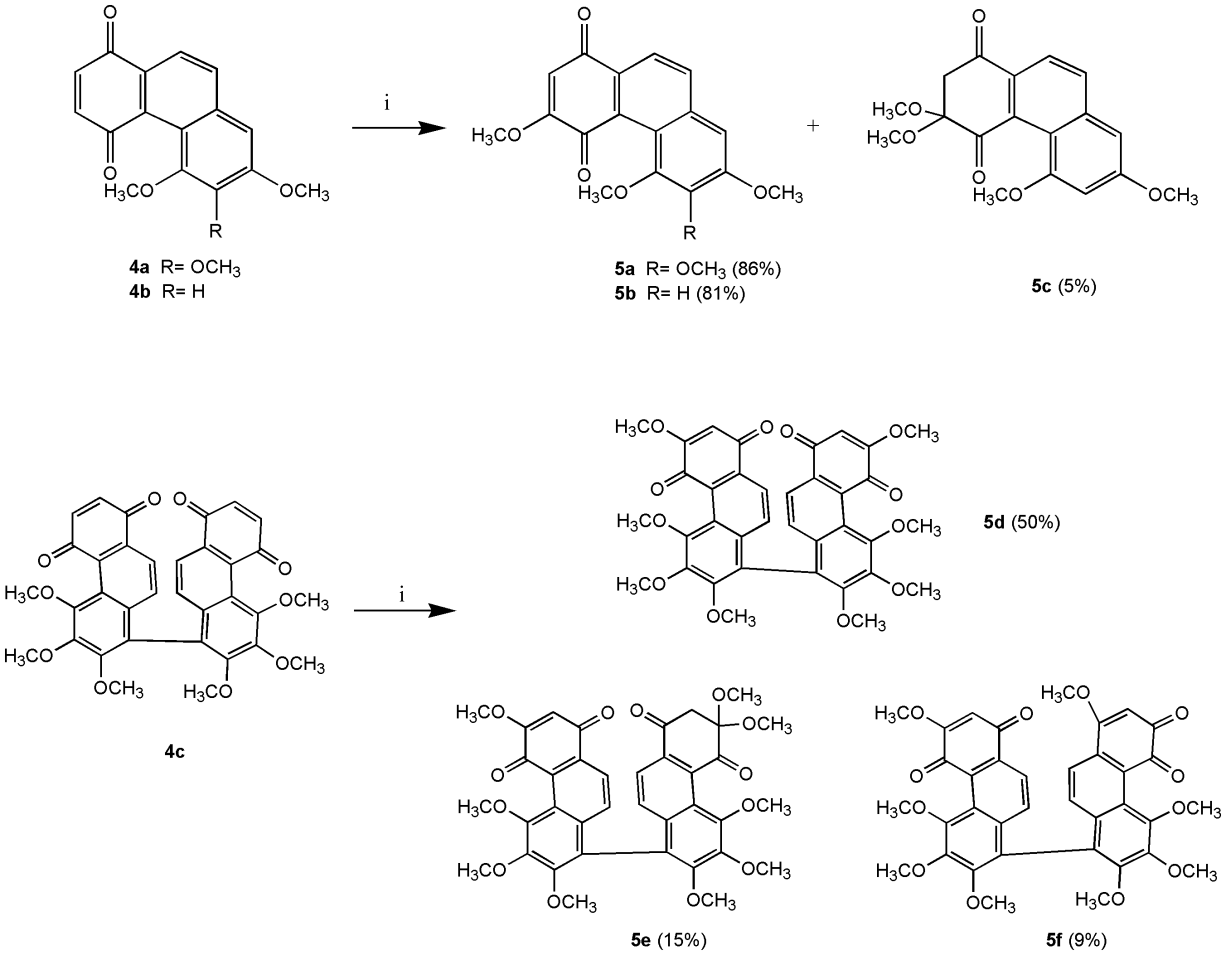
Synthesis of methoxy-phenanthrenequinones. Reagents and conditions: (i) MeOH, Fe_2_(SO_4_)_3_, PTSA, 70°C.

**Figure 5:**
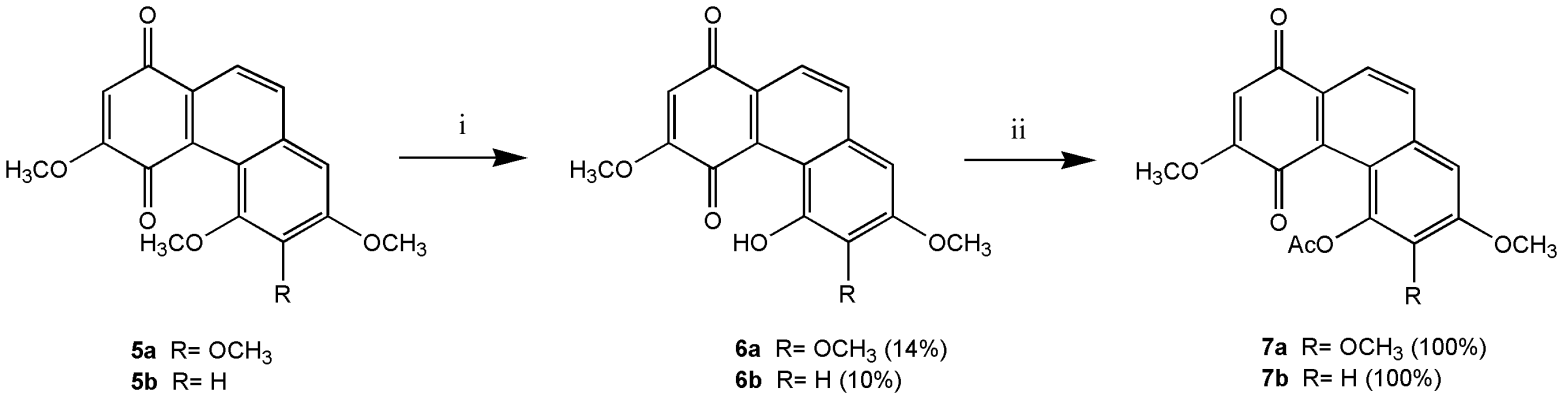
Selective demethylation and acetylation of phenanthrenequinones. Reagents and conditions: (i) TMSI, CH_2_Cl_2_, RT or 60°C. (ii) Ac_2_O, pyridine, RT.

**Figure 6:**
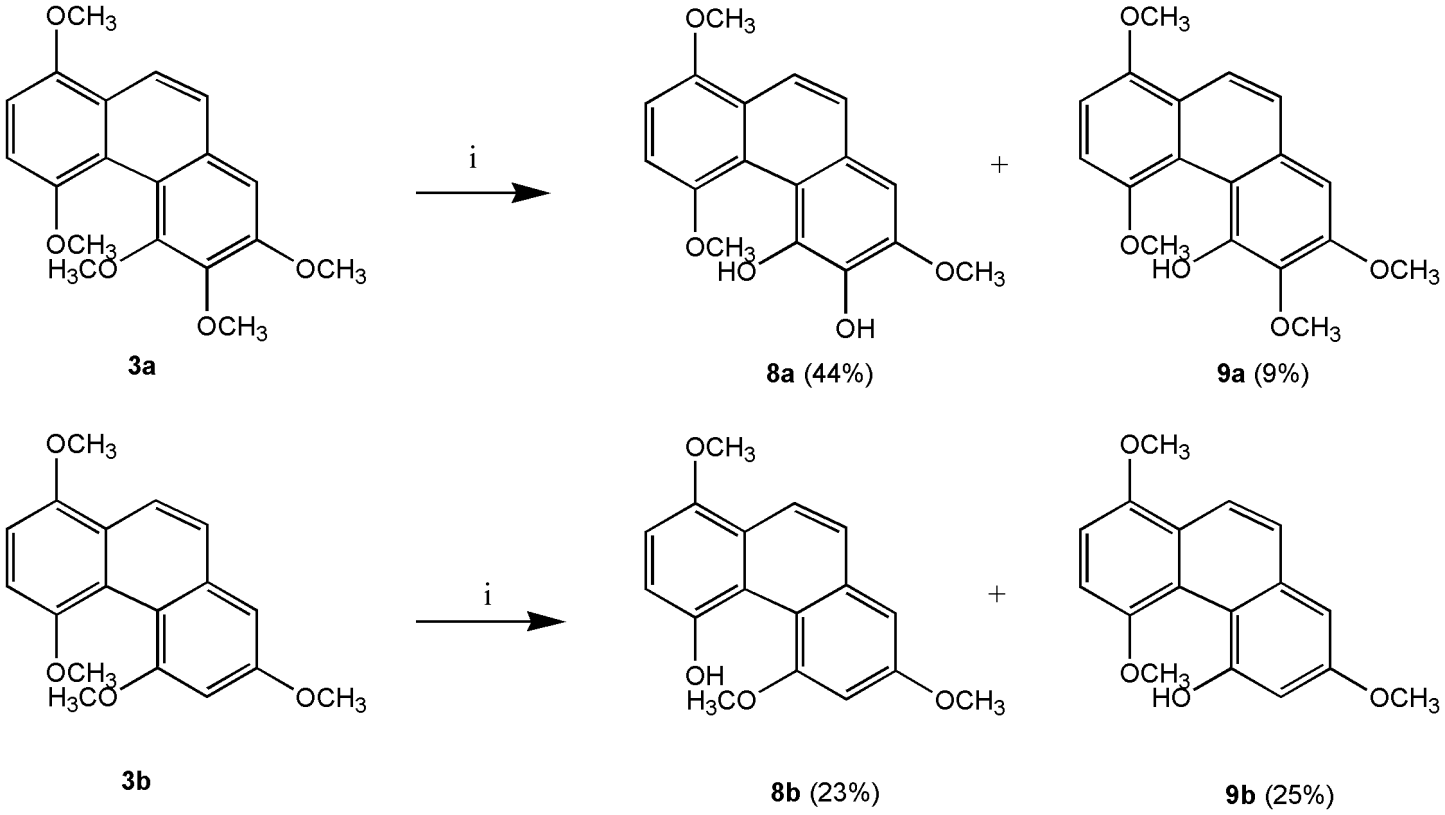
Demethylation of phenanthrenes. Reagents and conditions: (i) AlCl_3_, benzene, 70°C.

We modified the synthetic procedure of Dr. Kraus and his co-workers [12], [13] to synthesize all phenanthrene derivatives. As shown in Figure 3, 2-aldehyde-1,4-quinone was prepared by DDQ oxidation of commercially available 2,5-dihydroxybenzaldehyde. The quinone was coupled with 3,4,5-trimethoxytoluene and 3,5-dimethoxytoluene in the presence of 1 equivalent of trifluoroacetic acid to produce **1a** and **1b**, respectively. Compounds **1a** and **1b** were methylated with Me_2_SO_4_ in the presence of K_2_CO_3_ (acetone, 60°C, 5 h) to give the desired **2a** and **2b**. Cyclization of **2a** and **2b** with P_4_-*t*Bu (benzene, 110 or 140°C, 19–29 h) gave phenanthrenes **3a** and **3b**, which were oxidized with AgO (6 N HNO_3_, acetone, 50°C, 2–3 min) to phenanthrenequinone **4a**, **4b** and **4c**. Addition of methanol to **4a**, **4b** and **4c** catalyzed by ferric sulfate [14] gave **5a**–**f**, respectively (Figure 4). Compounds **7a** and **7b** were obtained by treatment of **5a** and **5b** with TMSI (CH_2_Cl_2_, RT or 60°C, monitored by TLC) to give calanquinone A (**6a**; **CA-1**) and denbinobin (**6b**), followed by treatment with Ac_2_O (pyridine, RT, overnight) to selectively remove the methyl group and incorporate an acetyl group at C-5, respectively (Figure 5). It is a characteristic feature of the angular arrangement of 1,4-phenanthrenequinones which led to remarkable selectivity in the cleavage of sterically hindered methyl ether at C-5 even in preference of that at C-3. However, applying TMSI to remove the methyl groups in phenanthrenes **3a** and **3b** was unsuccessful. Finally, cleavage of the methyl ether groups in **3a** and **3b** with AlCl_3_ generated compounds **8a** & **9a** and **8b** & **9b**, respectively (Figure 6). The excess AlCl_3_ regioselectively cleaved the methyl ethers only at C-4 and C-5 or C-6 in order to release the steric strain.

### Cytotoxicity

The cytotoxic assay of 11 naturally occurring and 19 synthesized phenanthrenes was carried out on a diverse set of human liver (HepG2 and Hep3B), oral (Ca9-22), lung (A549) and breast (MEA-MB-231 and MCF7) cancer cell lines, and a human fetal lung fibroblast (MRC-5) cell line (Tables 1 and 2). Doxorubicin was used as a positive control and an IC_50_>4 µg/mL was considered inactive.

**Table 1:** Cytotoxicity data of natural phenanthrenes isolated from *C. arisanensis*.

	IC_50_ (µg/mL)/Cell line						
Compd	HepG2	Hep3B	Ca9-22	A549	MCF-7	MDA-MB-231	MRC-5
**CA-1**	0.21±0.01	0.22±0.00	0.17±0.01	0.11±0.00	0.09±0.00	0.64±0.06	0.65±0.00
**CA-2**	14.47±0.43	11.94±0.55	14.64±0.11	18.50±0.29	11.90±0.08	>20	>20
**CA-3**	19.76±0.16	6.02±0.12	12.55±0.76	>20	13.25±0.00	11.90±0.02	>20
**CA-4**	>20	11.05±0.73	15.12±0.14	>20	14.30±0.18	19.83±0.45	>20
**CA-5**	17.80±0.40	9.87±0.09	12.17±0.03	>20	12.70±0.07	11.66±0.13	>20
**CA-6**	>20	11.50±0.76	14.56±0.31	>20	>20	>20	>20
**CA-7**	>20	>20	>20	>20	14.62±0.34	>20	>20
**CA-8**	>20	>20	>20	>20	>20	>20	>20
**CA-9**	>20	12.89±0.32	12.99±0.42	18.11±0.26	10.25±0.27	19.48±0.58	>20
**CA-10**	7.52±0.00	7.24±0.18	6.11±0.51	7.46±0.32	6.77±0.12	7.56±0.03	16.39±0.36
**CA-11**	7.17±0.35	6.28±0.26	5.86±0.06	7.40±0.01	4.84±0.89	7.42±0.03	13.17±0.33
**Doxo** a	0.22±0.04	0.49±0.00	0.16±0.02	0.81±0.04	0.67±0.03	0.74±0.01	1.94±0.02

a Doxorubicin (Doxo) was used as the positive control.

**Table 2:** Cytotoxicity data of synthesized phenanthrenes.

	IC_50_ (µg/mL)/Cell line						
Compd	HepG2	Hep3B	Ca9-22	A549	MCF-7	MDA-MB-231	MRC-5
**3a**	>20	15.55±0.08	>20	>20	>20	>20	>20
**3b**	>20	>20	18.44±0.11	>20	>20	>20	>20
**4a**	15.68±0.01	7.33±0.03	2.17±0.01	15.94±0.01	13.19±0.38	4.60±0.06	11.93±0.12
**4b**	18.64±0.14	12.10±0.33	3.45±0.03	>20	15.63±0.01	7.09±0.01	>20
**4c**	6.87±0.10	4.37±0.00	1.90±0.01	12.65±0.48	4.71±0.26	4.09±0.12	5.37±0.25
**5a**	4.65±0.07	4.67±0.81	5.99±0.24	11.04±0.44	14.29±0.50	>20	>20
**5b**	12.99±0.21	15.95±0.21	15.25±0.05	15.71±0.17	14.40±0.28	18.98±0.55	>20
**5c**	>20	13.78±0.38	9.07±0.09	11.99±2.18	11.10±0.02	19.11±0.43	>20
**5d**	1.49±0.01	6.66±0.13	4.36±0.03	9.30±0.48	13.78±0.06	14.76±0.25	>20
**5e**	1.24±0.08	7.38±0.02	6.21±0.12	7.49±0.07	9.40±0.00	6.74±0.05	8.11±0.25
**5f**	19.64±0.74	7.88±0.19	4.57±0.03	>20	14.77±0.21	4.74±0.02	>20
**6a**	0.08±0.00	0.19±0.01	0.59±0.01	0.14±0.02	0.20±0.00	0.89±0.01	0.60±0.05
**6b**	0.23±0.01	0.34±0.00	0.68±0.00	0.99±0.02	0.26±0.00	1.06±0.03	2.14±0.08
**7a**	11.30±0.14	0.36±0.14	0.84±0.05	0.60±0.01	0.16±0.00	1.13±0.05	1.14±0.01
**7b**	13.23±0.25	0.60±0.11	1.55±0.20	1.66±0.01	0.53±0.03	1.61±0.04	2.64±0.06
**8a**	19.38±0.56	5.77±0.63	3.91±0.07	>20	17.01±0.42	15.63±1.52	>20
**9a**	>20	>20	>20	>20	>20	>20	>20
**8b**	10.08±0.66	6.93±0.19	12.07±0.37	19.58±0.22	19.31±0.08	>20	>20
**9b**	>20	>20	>20	>20	>20	>20	>20
**Doxo** a	0.22±0.02	0.42±0.01	0.14±0.04	0.63±0.15	0.35±0.09	1.12±0.05	1.94±0.02

a Doxorubicin (Doxo) was used as the positive control.

Among the 11 naturally occurring compounds [2], calanquinone A (**CA-1**) (5-OH, 6-OCH_3_) and calanquinone B (**CA-2**) (5-OCH_3_, 6-OH) simply have reversed placements of the OH and one OCH_3_ group, but **CA-2** was much less potent than **CA-1** (Figure 2 and Table 1). The SAR results of **CA-1** and **CA-2** could possibly be explained by intramolecular hydrogen bonding between C = O (C-4) and OH (C-5) groups in 3-methoxy-1,4-PQs that may be a necessary moiety for cytotoxicity. To set up SAR correlations and identify active phenanthrene analogs, calanquinone A (**CA-1**) was selected as a lead compound for further studies.

Accordingly, 19 analogues including calanquinone A (**6a**; **CA-1**) were synthesized and tested in cytotoxicity assays. As shown in Table 2, calanquinone A (**6a**) and denbinobin (**6b**) exhibited significant potency against all cancer cell lines (IC_50_ 0.08–1.06 µg/mL). PQs **7a** and **7b** also showed very high potency against five cancer cell lines (IC_50_ 0.16–1.66 µg/mL), not including HepG2. Conversely and interestingly, PQs **5d** and **5e** were active only against the HepG2 cancer cell line with IC_50_ values of 1.49 and 1.24 µg/mL, respectively. PQs **4a**, **4b**, **4c** and phenanthrene **8a** displayed selective activity toward the Ca9-22 cancer cell line with IC_50_ values of 2.17, 3.45, 1.90 and 3.91 µg/mL, respectively. The SAR study of cytotoxicity suggested that the skeleton of 1,4-PQ is preferable to that of phenanthrene. To evaluate a potential SAR of the intramolecular hydrogen bond between C-4 and C-5, 3-methoxy-1,4-PQs **5a**–**b**, **6a**–**b** and **7a**–**b** were designed. Compounds **6a** and **6b**, with OH at C-5 and C = O at C-4, can form an intramolecular hydrogen bond. However, the hydrogen donors of **5a**–**b** and **7a**–**b** have been replaced with OCH_3_ and OAc groups, respectively. Among the six 3-methoxy-1,4-PQs, **6a** and **6b** exhibited the most significant potency, especially **6a** (IC_50_ 0.08–0.89 µg/mL). Compounds **5a** and **5b** showed marginal activities against all human cancer cell lines. Surprisingly, the new **7a** and **7b**, with OAc at C-5 and C = O at C-4, were active toward five human cancer cell lines (IC_50_ 0.16–1.66 µg/mL), but not HepG2. These data also represent the first time we have found this phenomenon in a cytotoxic assay of PQ derivatives. To expand upon the SAR study, all natural and synthesized compounds were used for the 3D pharmacophore model building.

### 3D Pharmacophore Modeling

To further identify the critical structural features of the phenanthrene analogs, 29 compounds (Figure 2) were used for pharmacophore modeling with Catalyst HypoGen. In this spatial aspect model, the phenanthrene structures and their cytotoxicity toward MCF-7 cancer cell line showed some interesting information.

**Figure 7:**
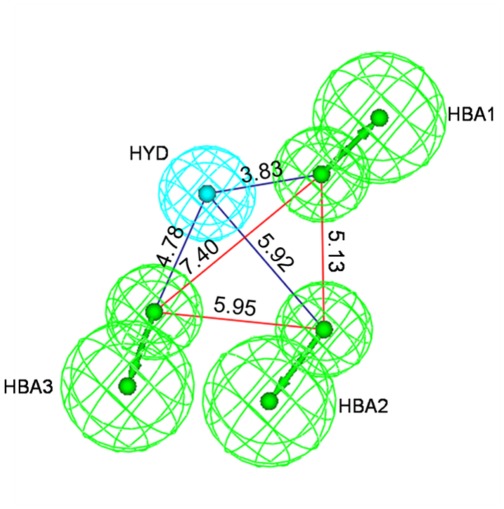
The mutual distances of the hydrogen bond triad and the distances between hydrogen bonds and hydrophobic group in the best HypoGen pharmacophore model. The pharmacophore features are colored with green, as are the hydrogen-bond acceptors (HBA1, HBA2 and HBA3). The hydrophobic aromatic feature (HYD) is denoted in cyan. Distances between features are in Ångström units.

The best pharmacophore model was established as a result of thirty runs with various parameters and characterized by a best correlation coefficient (0.931), the lowest total cost value (109.366), the highest cost difference (42.417), and the lowest RMS (0.790) (for details see Tables S1, S2 and Figure S1; Text S1). As shown in Figure 7, four essential features, three hydrogen bond acceptors (HBA1, HBA2 and HBA3) and one hydrophobic feature (HYD) were defined. All mutual distances of the four features can be measured. The distances between HBA1 and HBA2 or HBA3 were found to be 5.13 and 7.40 Å, respectively. The distances between HBA2 and HBA3 or HYD were found to be 5.95 and 5.92 Å, respectively. The distances between HYD and HBA1 or HBA3 were found to be 3.83 and 4.78 Å, respectively. The distance between HBA2 and HBA3 is especially critical for the MCF-7 cytotoxic effect in this model.

**Figure 8:**
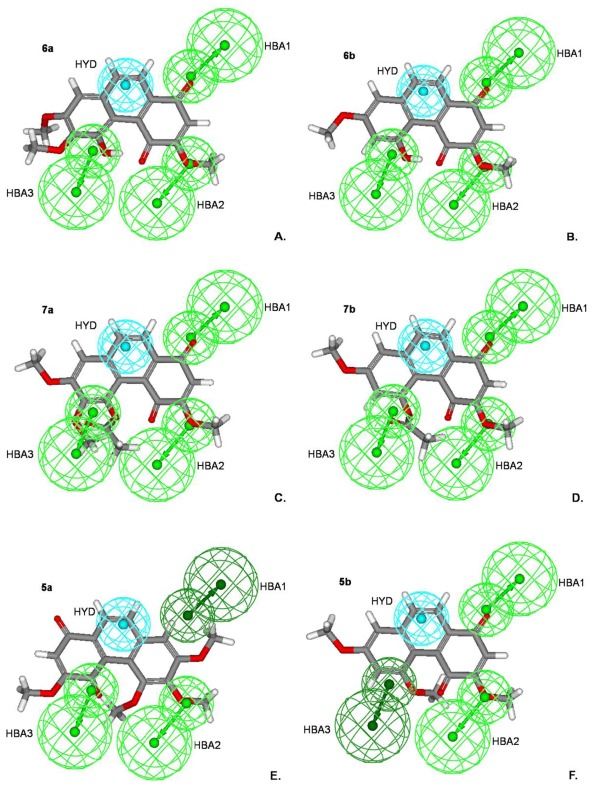
The best HypoGen pharmacophore model mapping onto calanquinone A (6a) and its derivatives. The light and dark green represent active and inactive features, respectively. The models mapped with the compounds **6a** (A), **6b** (B), **7a** (C), **7b** (D), **5a** (E) and **5b** (F) are shown here. Pharmacophore features are colored as in Figure 7.

The mappings of the best model with all compounds show fit values ranging from 6.18 to 8.57 (Table S3; Text S1). Calanquinone A (**6a**) mapped to the best hypothesis model with the fit value of 8.57 reveals significant features in Figure 8A. Obviously, the HBA1 links to the carbonyl group of quinone ring at C-1, HBA2 links to the oxygen atom of the methoxyl group at C-3, HBA3 links to the oxygen atom of the hydroxyl group at C-5, and HYD aligns to the aromatic ring (B-ring). Compound **6b** has a similar alignment as **6a**, with a high fit value of 7.95 (Figure 8B and Table S3). As shown in Figures 8C and 8D, **7a and 7b**, which are highly toxic to MCF-7 cells, also match against all features of the best hypothesis in which **7a** was originally designed to remove the intramolecular hydrogen bond and was previously speculated to be less cytotoxic. The main difference in structure between **7a**–**b** and **6a**–**b** is the carbonyl group of the acetoxyl substituent at C-5. Consequently, the conserved distance between HBA2 and HBA3 explains why **7a** and **7b**, with OCOCH_3_ at C-5 and C = O at C-4 but without the same intramolecular hydrogen bonds as **6a** and **6b**, can still possess significant cytotoxicity, in contrast to our previous speculation. For less MCF-7 cytotoxic compounds, mismatching one hydrogen bond in the triad and thus disrupting the structures of **5a** and **5b** results in moderate inhibition, one order higher in µg/mL. The oxygen atom of the methoxyl group at C-6, the carbonyl group at the C-4 position, and the aromatic atom at C-9 of **5a** fit into the HBA2, HBA3 and HYD, respectively. However, they do not fit into HBA1 (Figure 8E). Also, the carbonyl group at C-1, the oxygen atom of the methoxyl group at C-3, and the aromatic atom at C-9 of **5b** match against HBA1, HBA2 and HYD features, but are not linked to HBA3 (Figure 8F).

Thus, HBA1, 2 and 3 complete the triad of the hydrogen acceptor feature and clearly explain the MCF-7 cytotoxic variation of **5a**–**b**, **6a**–**b** and **7a**–**b**. In addition, the hydrophobic feature HYD indicates a pharmacophore anchor for a three-ring core as in phenanthrene and PQ derivatives in the series. The hydrophobic feature links to all compounds and the HBA feature always links to the carbonyl group at C-1 or C-4 of the quinone ring in all PQs. As a whole, a pharmacophore and explicit SAR were established herein.

### ChemGPS-NP Analysis of Calanquinone A (6a) and Denbinobin (6b)

**Figure 9:**
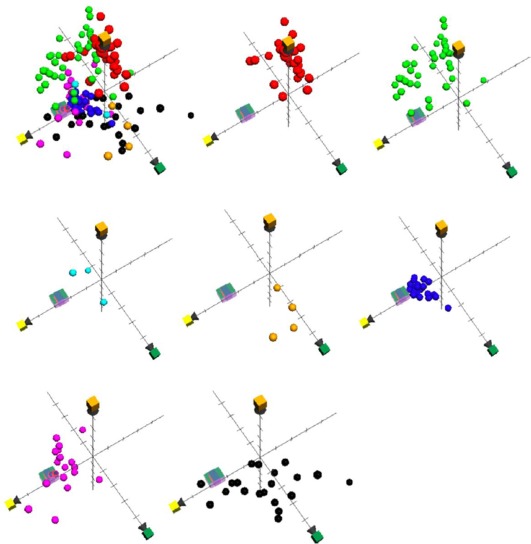
ChemGPS-NP analysis of calanquinone A (6a) and denbinobin (6b). Score plot of the three dimensions (principal components 2–4) consisting of PC2 (yellow; aromaticity etc.), PC3 (green; lipophilicity etc.) and PC4 (orange; flexibility/rigidity), from analysis of most potent compounds **6a** and **6b** as medium seagreen cubes in the ChemGPS-NP model addressed by Rosén et al. in 2009 for prediction of MOA. A reference set of known anticancer agents includes alkylating agents (red), anti-metabolites (lime), proteasome inhibitions (cyan), tyrosine kinase inhibitors (orange), topoisomerase I (blue), topoisomerase II (magenta), and tubulin inhibitors (black).

ChemGPS-NP (chemical global positioning system for natural products) is a computational model based on principal component analysis of physical-chemical properties. Such properties can be estimated directly from structure data, and by performing score prediction in the ChemGPS-NP model, this provides a versatile tool for charting and navigating the biologically relevant chemical space [15]. In a previous study ChemGPS-NP has successfully been used to chart a set of known anticancer agents with different cytotoxic mechanisms. The resulting map has been used as a tool to predict the anticancer Mode of Action (MOA) for new and previously unstudied lead compound [16]. As shown in Figure 9, the two most potent cytotoxic compounds, calanquinone A (**6a**) and denbinobin (**6b**), were predicted in the model. Evaluating their resulting position on the chemical space map, it can be concluded that these phenanthrene derivatives do not unambiguously belong to any of the well defined groups representing alkylating agents, antimetabolites, proteasome inhibitions, tyrosine kinase inhibitors, topoisomerase I, and tubulin inhibitors except topoisomerase II inhibitors. The preliminary result of this ChemGPS-NP analysis indicates that calanquinone A (**6a**) and denbinobin (**6b**) might be members of a topoisomerase II inhibitor, which however, still remains to be further elucidated.

### Topoisomerase II Assay

**Figure 10:**
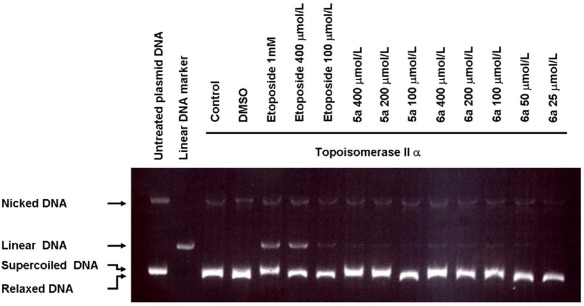
Topoisomerase II DNA cleavage assay. An in vitro assay was used to assess the effect of compounds **6a** and **5a** on the DNA cleavage activity of human TopoII. Etoposide was the positive control. Control lane: TopoIIα + plasmid DNA. DMSO lane: TopoIIα + plasmid DNA + DMSO.

From the ChemGPS-NP analysis, it seems that the MOA for 3-methoxy-1,4-PQs might processes cytotoxicity as a topoisomerase II inhibitor. In the previous study [17], 1,4-benzoquinone has been found to poison human topoisomerase IIα. According to these results, we chose the most potential calanquinone A (**6a**) and its moderate compound **5a** to test the DNA cleavage assay, in which known etoposide (VP-16) was used as the positive control. As shown in Figure 10, compound **6a** showed the inhibition on Topo IIα in the result of the appearance of supercoided DNA instead of the relaxed one at the concentration of 100 µmol/L. Additionally, compound **5a** also had the similar effect at higher concentration (200 µmol/L). Moreover, both compounds induced the formation of linear DNA, suggesting that they could possibly trap Topo IIα into DNA cleavage complex. Our data proved PQs inhibit hTopoII in vitro with inducing DNA strand breaks and protein covalently bound to DNA, ultimately leading to cell cycle arrest and death.

### Conclusion

In summary, a series of phenanthrene derivatives, including the new derivatives (**3a**, **4c**, **5c**–**f**, **7a**, **8a**–**b** and **9a**–**b**), were synthesized in this investigation. On the basis of our SAR studies, 3-methoxy-1,4-PQs **6a** (calanquinone A), **6b** (denbinobin), **7a** (5-OAc-calanquinone A) and **7b** (5-OAc-denbinobin) were identified as highly potent cytotoxic agents.

A best ligand-based pharmacophore model against the MCF-7 cancer cell line was successfully established. It explains the SAR of 3-methoxy-1,4-PQs **5a**–**b** (5-OCH_3_), **6a**–**b** (5-OH) and **7a**–**b** (5-OCOCH_3_) in a spatial aspect model. Highly active **6a**, **6b**, **7a** and **7b** possess three hydrogen bond acceptors forming a hydrogen bond triad combined with one hydrophobic group as a pharmacophore that can interact with a potential target. The revealed pharmacophore model provides a bona fide basis for further design and synthesis of promising phenanthrene structures in vitro to study their anti-breast cancer properties. On the basis of ChemGPS-NP prediction and TopoII assay assessment, 1,4-PQs were suggested as the topoisomerase II inhibitors. This is the first time to apply ChemGPS-NP to previously untested cytotoxic compounds for MOA prediction. In the future, ChemGPS-NP could be used to effectively find the most possible MOA in the new drug discovery, as suggested by Rosén and co-workers [16].

Overall, our data demonstrate that PQs could be promising lead compounds for the further development of anti-cancer.

## Materials and Methods

### General

Melting points were determined on a Yanaco® digital micro melting point apparatus model MP-500D without correction. NMR spectra were recorded on Varian Unity-plus 400 MHz FT-NMR and Varian Mercury-plus 400 MHz FT-NMR instruments. Chemical shift (δ) values are in ppm (parts per million) with CDCl_3_ as the internal standard, and coupling constants (*J*) are in Hz. HRESI-MS and ESI-MS measurements were performed on a Bruker Daltonics APEX II 30e mass spectrometer. TLC was performed on Kieselgel 60, F 254 (0.25 nm, Merck), and spots were viewed under ultraviolet light at 254 and 356 nm. For column chromatography, silica gel (Kieselgel 60, 70–230, and 230–400 mesh, Merck) and a Biotage® SP system apparatus were used.

### Cytotoxicity ASSAY

Compounds were tested against human liver (HepG2 and Hep3B), oral (Ca9-22), lung (A549), breast (MEA-MB-231 and MCF7) cancer cell lines, and the human fetal lung fibroblast (MRC-5) cell line using an established colorimetric MTT assay protocol [18]. The absorbance was measured at 550 nm using a microplate reader. The IC_50_ is the concentration of agent that reduced cell growth by 50% under the experimental conditions.

### 3D Pharmacophore Model

The pharmacophore modeling with Catalyst HypoGen was performed via Discovery Studio 2.1 (Accelrys, San Diego, CA, USA) [19]. Twenty-nine phenanthrene derivatives were collected from the natural plant, *C. arisanensis,* and from chemical synthesis (Figure 2). Cytotoxicity against MCF-7 cells was determined by the MTT assay and the concentration (µg/mL) of test compound which inhibited 50% of the cancer cells (IC_50_) was used in the generation of the pharmacophore model. An IC_50_ value of >20 µg/mL was defined as 20 µg/mL. All experimental IC_50_ values spanned about 2–3 orders of magnitude from 0.09 to 20 µg/mL. The 2D/3D structures of compounds were generated using ChemBioOffice 2008 (Cambridge Scientic Computing, Cambridge, Massachusetts, USA) and then optimized in a Dreiding force field. The conformational ensemble of each compound was generated using the best conformational analysis method based on a CHARMM force field with a 20 kcal/mol energy threshold above the global minimum. A maximum limit of 255 conformations was used to cover maximum conformational space. The best 3D arrangements of chemical functionalities should explain the activity variations among the 29 compounds. Thirty runs with different parameters were performed for the best pharmacophore hypothesis. Four chemical features, including hydrogen-bond acceptor (HBA), hydrogen-bond donor (HBD), hydrophobic (HYD), and aromatic ring (AR) features, were also tested during the building of pharmacophore hypotheses (Table S1; Text S1). The best hypotheses were selected via a correlation and a cost analysis in Catalyst HypoGen.

Three costs including the total cost (the sum of weight, error and configuration cost), the null cost and the fixed cost will be evaluated. A total cost that is similar to the fixed cost and far from the null cost indicates statistically significant pharmacophore hypotheses. A difference between the total cost and null cost ranging from 40 to 60 indicates a true correlation of the pharmacophore hypothesis with 75–90% high probability. The true correlation represents <50% probability when it is less than 40. Generally, the configuration cost should be smaller than 17 in a standard HypoGen model. According to the total cost (109.366), fixed cost (99.558), null cost (151.783), RMS value (0.790), and correlation coefficient (0.931) (Table S2), the best pharmacophore hypothesis, run 22, containing three hydrogen-bond acceptors (HBA1, HBA2, HBA3) and one hydrophobic feature (HYD) was selected (Figure 7).

### ChemGPS-NP

The PCA-based model ChemGPS-NP (http://chemgps.bmc.uu.se) is a tool for navigation in biologically relevant chemical space. It has eight principal components (PC; dimensions), derived from 35 molecular descriptors describing physical-chemical properties such as size, shape, polarizability, lipophilicity, polarity, flexibility, rigidity, and hydrogen bond capacity for a reference set of compounds. The ChemGPS-NP descriptors were calculated for compounds **6a** and **6b** on the basis of their structure information as simplified molecular input line entry specification (SMILES) using the software DRAGON Professional. Compounds **6a** and **6b** were then mapped onto ChemGPS-NP using interpolation in terms of PCA score together with a reference set of known anticancer agents with previously studied Mode of Action (MOA) (Anticancer Agent Mechanism Database; http://dtp.nci.nih.gov/docs/cancer/searches/standard_mechanism.html). Principal component and PCA score prediction were calculated employing the software SIMCA-P+, with the training set ChemGPS-NP. Prior to PCA determination, all data were centered and scaled to unit variance [16].

### Topoisomeras II Assay

Topoisomerase II assay was performed by using a Topo II Drug Screening Kit (TopoGEN, Inc.). In brief, 0.1 µg of pHOT plasmid DNA was incubated with 2 units of topoisomerase IIα in 20 µL assay buffer at 37°C for 40 min in the presence of tested compounds (**6a**, **5a**) and control drug, etoposide, respectively. 2 µL of 10% SDS and 2.5 µL of 10 mg/mL proteinase K were added into the reaction sample and then incubated for 30 min at 37°C to digest topoisomerase IIα. The samples were mixed with 2 µL of loading buffer and cleaned up by adding an equal volume of phenol:chloroform:isoamyl alcohol (25∶24∶1) according to the description. The sample was mixed by vortex and centrifuge for 10 sec. An aliquot (10 µL) of the upper aqueous part was analyzed by electrophoresis with 2% agarose gel containing 0.5 µg/mL of ethidium bromide [20].

#### 3,6-Dihydroxy-2′,3′,4′-trimethoxy-6′-methyl-biphenyl-2-carbaldehyde (1a)

2,5-dihydroxybenzaldehyde (3.01 g, 21.79 mmol) and DDQ (9.18 g, 40.42 mmol) were dissolved in dry benzene. The mixture was stirred at RT overnight, and the benzene was then evaporated. The crude product reacted with equimolar amounts of 3,4,5-trimethoxytoluene and trifluoroacetic acid in Et_2_O at RT for 24 h or for an extended reaction time to obtain the best yield. The crude product was poured onto ice water and extracted three times with EtOAc. The EtOAc layer was washed once with brine and dried with Na_2_SO_4_. The product was chromatographed on silica gel and eluted with CH_2_Cl_2_ to give compound **1a** (2.14 g, 31.16%). ^1^H NMR (CDCl_3_): δ 2.01 (s, 3 H), 3.65 (s, 3 H), 3.87 (s, 3 H), 3.91 (s, 3 H), 4.65 (s, OH), 6.68 (s, 1 H), 6.95 (d, 1 H, *J* = 8.8 Hz), 7.23 (d, 1 H, *J* = 8.8 Hz), 9.45 (s, 1 H), 11.38 (s, OH); ^13^C NMR (CDCl_3_): δ 19.9, 56.0, 61.0, 61.1, 109.7, 116.2, 117.8, 118.2, 125.2, 125.5, 133.9, 140.5, 145.6, 152.2, 154.4, 156.9, 196.7.

#### 3,6-Dihydroxy-2′,4′-dimethoxy-6′-methyl-biphenyl-2-carbaldehyde (1b)

2,5-dihydroxybenzaldehyde (771.46 mg, 5.59 mmol), DDQ (2.54 g, 11.17 mmol), 3,5-dimethoxytoluene (850 mg, 5.59 mmol), and trifluoroacetic acid were used with the method described for **1a** to yield **1b** (842.40 mg, 52.69%). ^1^H NMR (CDCl_3_): δ 1.91 (s, 3 H), 3.62 (s, 3 H), 3.72 (s, 3 H), 3.85 (s, 3 H), 3.88 (s, 3 H), 3.91 (s, 3 H), 6.58 (s, 1 H), 6.98 (d, 1 H, *J* = 8.8 Hz), 7.15 (d, 1 H, *J* = 8.8 Hz), 9.98 (s, 1 H);^ 13^C NMR (CDCl_3_): δ 19.9, 55.8, 56.1, 56.4, 60.5, 60.9, 108.6, 111.4, 117.0, 120.7, 124.5, 130.4, 132.1, 139.8, 151.0, 151.0, 152.8, 154.6, 191.6.

#### 3,6,2′,3′,4′-Pentamethoxy-6′-methyl-biphenyl-2-carbaldehyde (2a)

The hydroquinone **1a** (2.15 g, 6.76 mmol) and K_2_CO_3_ (10.25 g, 74.16 mmol) were dissolved in acetone and then dimethyl sulfate (2.13 g, 16.89 mmol) was added. The mixture was stirred at reflux for 5 h, and the K_2_CO_3_ was then removed by filtration through Celite. The crude product was chromatographed on silica gel and eluted with EtOAc/*n*-hexane (1∶2) to yield **2a** (1.88 g, 80.57%). ^1^H NMR (CDCl_3_): δ 2.03 (s, 3 H), 3.70 (s, 3 H), 3.86 (s, 3 H), 4.58 (s, OH), 6.44 (d, 1 H, *J* = 2.4 Hz), 6.52 (d, 1 H, *J* = 2.4 Hz), 6.92 (d, 1 H, *J* = 8.8 Hz), 7.21 (d, 1 H, *J* = 8.8 Hz), 9.39 (s, 1 H), 11.35 (s, OH); ^13^C NMR (CDCl_3_): δ 20.2, 55.4, 55.7, 96.5, 107.2, 110.5, 117.9, 125.2, 125.8, 140.8, 145.6, 156.8, 158.0, 159.0, 161.6, 197.0.

#### 3,6,2′,4′-Tetramethoxy-6′-methyl-biphenyl-2-carbaldehyde (2b)

Compound **1b** (8.57 mg, 29.76 mmol), K_2_CO_3_ (41 g, 296.65 mmol), and dimethyl sulfate (11.20 g, 88.80 mmol) were used with the method described for **2a** to yield **2b** (6.23 g, 66.28%). ^1^H NMR (CDCl_3_): δ 1.96 (s, 3 H), 3.65 (s, 3 H), 3.69 (s, 3 H), 3.83 (s, 3 H), 3.91 (s, 3 H), 6.37 (d, 1 H, *J* = 2.4 Hz), 6.44 (d, 1 H, *J* = 2.4 Hz), 6.96 (d, 1 H, *J* = 8.8 Hz), 7.14 (d, 1 H, *J* = 8.8 Hz), 9.85 (s, 1 H); ^13^C NMR (CDCl_3_): δ 20.2, 55.2, 55.7, 56.1, 56.7, 96.0, 106.4, 111.3, 115.3, 117.3, 124.7, 131.0, 138.9, 151.2, 154.3, 157.8, 160.2, 192.0.

#### 1,4,5,6,7-Pentamethoxy-phenanthrene (3a)

Compound **2a** (1.18 g, 3.41 mmol) and P_4_-*t*Bu (1 M, 5.50 mL, 5.45 mmol) were placed under N_2_ in benzene and heated to 140°C for 29 h. The solvent was evaporated and the crude product was chromatographed on silica gel eluting with EtOAc/*n*-hexane (1∶2) to yield **3a** (1.06 g, 95.04%). Pale yellowish gum; ^1^H NMR (CDCl_3_): δ 3.71 (s, 3 H), 3.94 (s, 3 H), 3.99 (s, 3 H), 4.00 (s, 3 H), 4.01 (s, 3 H), 6.91 (d, 1 H, *J = *8.8 Hz), 6.98 (d, 1 H, *J = *8.8 Hz), 7.00 (s, 1 H), 7.52 (d, 1 H, *J = *8.8 Hz), 8.01 (d, 1 H, *J = *8.8 Hz); ^13^C NMR (CDCl_3_): δ 56.0, 56.2, 56.5, 60.9, 61.3, 103.09, 105.8, 107.9, 116.8, 119.7, 120.6, 124.3, 126.0, 130.6, 142.2, 149.3, 151.7, 152.5, 152.7; HRESIMS *m/z* 351.1207 (calculated for C_19_H_20_O_5_Na, 351.1209).

#### 1,4,5,7-Tetramethoxy-phenanthrene (3b)

Compound **2b** (2.81 g, 8.89 mmol) and P_4_-*t*Bu (1 M, 11.50 mL, 11.56 mmol) were placed under N_2_ in benzene and heated to 110°C for 19 h. The solvent was evaporated and the crude product was chromatographed on silica gel and eluted with EtOAc/*n*-hexane (1∶7) to yield **3b** (3.59 g, 62.93%). Pale orange gum; ^1^H NMR (CDCl_3_): δ 3.95 (s, 3 H), 3.96 (s, 3 H), 3.98 (s, 3 H), 4.00 (s, 3 H), 6.72 (d, 1 H, *J = *2.4 Hz), 6.85 (d, 1 H, *J = *2.4 Hz), 6.90 (d, 1 H, *J = *8.4 Hz), 7.01 (d, 1 H, *J = *8.4 Hz), 7.53 (d, 1 H, *J = *8.8 Hz), 8.07 (d, 1 H, *J = *8.8 Hz); ^13^C NMR (CDCl_3_): δ 55.4, 55.8, 56.2, 56.3, 98.9, 99.8, 105.5, 108.3, 114.1, 120.7, 121.0, 124.3, 126.0, 135.5, 149.4, 151.5, 158.9, 159.1; ESIMS *m/z* 321 [M+Na]^+^.

#### 5,6,7-Trimethoxy-1,4-phenanthrenequinone (4a) and 5,6,7,5′,6′,7′-hexamethoxy-[8,8′]bi-1,4-phenanthrenequinone (4c)

Compound **3a** (1.46 g, 4.45 mmol) and AgO (2.20 g, 17.76 mmol) were dissolved in acetone. Oxidation was initiated by addition of 3.70 mL of 6 N HNO_3_. The reaction was allowed to stir at 50°C until the grey suspension disappeared (about 2–3 min). The reaction was quenched immediately with H_2_O and CH_2_Cl_2_. The CH_2_Cl_2_ layer was dried over Na_2_SO_4_ and chromatographed on silica gel and eluted with EtOAc/*n*-hexane (1∶3) to yield **4a** (385.46 mg, 29.76%) and **4c** (393.85 mg, 14.93%).

Data for **4a**: Red solid; mp 134°C;^ 1^H NMR (CDCl_3_): δ 3.96 (s, 3 H), 4.01 (s, 3 H), 4.04 (s, 3 H), 6.81 (d, 1 H, *J = *10.4 Hz), 6.96 (s, 1 H), 7.05 (d, 1 H, *J = *10.4 Hz), 7.86 (d, 1 H, *J = *8.4 Hz), 7.97 (d, 1 H, *J = *8.4 Hz); ^13^C NMR (CDCl_3_): δ 56.1, 60.9, 61.2, 102.7, 120.2, 121.4, 130.8, 131.7, 133.3, 134.7, 135.2, 140.3, 143.8, 150.3, 155.7, 184.8, 186.7; ESIMS *m/z* 321 [M+Na]^+^.

Data for **4c**: Red solid; mp 100°C; ^1^H NMR (CDCl_3_): δ 3.71 (s, 6 H), 3.99 (s, 6 H), 4.19 (s, 6 H), 6.83 (d, 2 H, *J = *10.0 Hz), 7.11 (d, 2 H, *J = *10.0 Hz), 7.30 (d, 2 H, *J = *8.4 Hz), 7.78 (d, 1 H, *J = *8.4 Hz); ^13^C NMR (CDCl_3_): δ 60.8, 61.1, 61.4, 119.6, 121.5, 122.1, 130.2, 131.5, 133.7, 134.0, 135.3, 140.4, 146.9, 151.1, 154.6, 184.7, 187.0; HRESIMS *m/z* 617.1421 (calcd for C_34_H_26_O_10_Na, 617.1424).

#### 5,7-Dimethoxy-1,4-phenanthrenequinone (4b)

Compound **3b** (30.45 mg, 0.11 mmol), AgO (48.41 mg, 0.39 mmol), and 0.08 mL 6 N HNO_3_ were used with the method described for **4a** and **4c**. The crude product was chromatographed on silica gel and eluted with EtOAc/*n*-hexane (1∶7) to yield **4b** (8.14 mg, 28.80%). Red needles; mp 121°C; ^1^H NMR (CDCl_3_): δ 3.92 (s, 3 H), 3.94 (s, 3 H), 6.69 (d, 1 H, *J = *2.4 Hz), 6.76 (d, 1 H, *J = *2.4 Hz), 6.78 (d, 1 H, *J = *10.4 Hz), 7.03 (d, 1 H, *J = *10.4 Hz), 7.85 (d, 1 H, *J = *8.8 Hz), 8.01 (d, 1 H, *J = *8.8 Hz); ^13^C NMR (CDCl_3_): δ 55.6, 55.9, 99.1, 101.9, 116.9, 122.6, 130.4, 131.7, 134.1, 135.0, 139.4, 140.3, 158.3, 161.1, 184.8, 186.7; ESIMS *m/z* 291 [M+Na]^+^.

#### 3,5,6,7-Tetramethoxy-1,4-phenanthrenequinone (5a)

*p*-Toluenesulfonic acid (126.70 mg, 0.67 mmol) and ferric sulfate (276.31 mg, 0.69 mmol) were added into the solution of **4a** (97.80 mg, 0.33 mmol) in 23 mL MeOH [14]. The mixture was heated at 70°C for 2 h and poured into water as well as extracted with CH_2_Cl_2_. The CH_2_Cl_2_ layer was dried over Na_2_SO_4_ and chromatographed on silica gel and eluted with CH_2_Cl_2_ to yield **5a** (93.09 mg, 86.48%). Yellow solid; mp 174°C; ^1^H NMR (CDCl_3_): δ 3.93 (s, 3 H), 3.94 (s, 3 H), 4.01 (s, 3 H), 4.08 (s, 3 H), 6.02 (s, 1 H), 6.96 (s, 1 H), 7.87 (d, 1 H, *J = *8.4 Hz), 8.00 (d, 1 H, *J = *8.4 Hz); ^13^C NMR (CDCl_3_): δ 56.0, 56.5, 60.8, 61.1, 102.6, 106.3, 120.3, 121.4, 131.2, 132.1, 132.2, 134.4, 143.8, 150.1, 155.4, 163.0, 182.0, 184.7; ESIMS *m/z* 351 [M+Na]^+^.

#### 3,5,7-Trimethoxy-1,4-phenanthrenequinone (5b) and 3,3,5,7-tetramethoxy-2,3-dihydro-1,4-phenanthrenequinone (5c)

Compound **4b** (202.92 mg, 0.76 mmol), *p*-toluenesulfonic acid (295 mg, 1.55 mmol), ferric sulfate (618 mg, 1.55 mmol) and 40 mL MeOH were used with the method described for **5a**. The crude product was chromatographed on silica gel and eluted with CH_2_Cl_2_ to yield **5b** (183.71 mg, 81.42%) and **5c** (11.51 mg, 4.60%).

Data for **5b**: Orange solid; mp 179°C; ^1^H NMR (CDCl_3_): δ 3.92 (s, 3 H), 3.93 (s, 6 H), 5.99 (s, 1 H), 6.69 (d, 1 H, *J = *2.4 Hz), 6.76 (d, 1 H, *J = *2.4 Hz), 7.87 (d, 1 H, *J = *8.4 Hz), 8.04 (d, 1 H, *J = *8.4 Hz); ^13^C NMR (CDCl_3_): δ 55.6, 55.9, 56.5, 99.1, 101.9, 106.2, 116.9, 122.6, 130.8, 132.3, 132.7, 139.0, 158.1, 160.8, 162.9, 181.8, 184.6; ESIMS *m/z* 321 [M+Na]^+^.

Data for **5c**: Pale yellowish solid; mp 149°C; ^1^H NMR (CDCl_3_): δ 3.29 (s, 2 H), 3.44 (s, 6 H), 3.92 (s, 3 H), 3.95 (s, 3 H), 6.67 (d, 1 H, *J* = 2.4 Hz), 6.81 (d, 1 H, *J* = 2.4 Hz), 7.84 (d, 1 H, *J* = 8.8 Hz), 7.99 (d, 1 H, *J* = 8.8 Hz); ^13^C NMR (CDCl_3_): δ 48.8, 50.3, 50.3, 55.6, 55.7, 99.5, 101.2, 101.5, 116.7, 123.2, 131.0, 132.3, 137.5, 139.6, 157.6, 161.4, 191.8, 196.9; HRESIMS *m/z* 353.0999 (calculated for C_18_H_18_O_6_Na, 353.1001).

#### 3,5,6,7,3′,5′,6,7′-Octamethoxy-[8,8′]bi-1,4,1′,4′-phenanthrenequinone (5d), 3,5,6,7,3′,3′,5′,6,7′-nonamethoxy-2′3′-dihydro-[8,8′]bi-1,4,1′,4′-phenanthrenequinone (5e), and 3,5,6,7,1′,5′,6′,7′-octamethoxy-[8,8′]bi-1,4,5′,6′-phenanthrenequinone (5f)

Compound **4c** (196.33 mg, 0.33 mmol), *p*-toluenesulfonic acid (277.8 mg, 1.46 mmol), ferric sulfate (531.90 mg, 1.33 mmol) and 20 mL MeOH were used with the method described for **5a**. The crude product was chromatographed on silica gel and eluted with EtOAc/*n*-hexane (1∶2) to yield **5d** (107.54 mg, 49.70%), **5e** (33.57 mg, 14.80%) and **5f** (20.32 mg, 9.40%).

Data for **5d**: Orange solid; mp 268°C; ^1^H NMR (CDCl_3_): δ 3.71 (s, 6 H), 3.97 (s, 6 H), 3.98 (s, 6 H), 4.22 (s, 6 H), 6.04 (s, 2 H), 7.31 (d, 1 H, *J = *8.8 Hz), 7.81 (d, 1 H, *J = *8.8 Hz); ^13^C NMR (CDCl_3_): δ 56.7, 60.7, 61.1, 61.4, 106.4, 119.7, 121.5, 122.2, 130.8, 132.0, 132.4, 133.8, 146.9, 150.8, 154.3, 163.0, 182.3, 184.4; HRESIMS *m/z* 677.1639 (calcd for C_36_H_30_O_12_Na, 677.1635).

Data for **5e**: Orange solid; mp 124°C; ^1^H NMR (CDCl_3_): δ 3.32 (s, 2 H), 3.50 (s, 3 H), 3.51 (s, 3 H), 3.69 (s, 3 H), 3.71 (s, 3 H), 3.96 (s, 3 H), 3.97 (s, 6 H), 4.20 (s, 3 H), 4.22 (s, 3 H), 6.04 (s, 1 H), 7.24 (d, 1 H, *J = *8.8 Hz), 7.30 (d, 1 H, *J = *8.8 Hz), 7.72 (d, 1 H, *J = *8.8 Hz), 7.81 (d, 1 H, *J = *8.8 Hz); ^13^C NMR (CDCl_3_): δ 49.0, 50.4, 50.5, 56.7, 60.5, 60.7, 60.8, 61.1, 61.2, 61.3, 101.7, 106.4, 119.6, 120.0, 121.5, 122.0, 122.2, 122.3, 129.3, 130.8, 132.0, 132.4, 133.3, 133.7, 134.1, 137.5, 146.2, 146.9, 150.3, 150.8, 154.3, 154.9, 163.0, 182.3, 184.4, 191.8, 197.7; HRESIMS *m/z* 709.1901 (calculated for C_37_H_34_O_13_Na, 709.1897).

Data for **5f**: Orange solid; mp 146°C; ^1^H NMR (CDCl_3_): δ 3.69 (s, 3 H), 3.70 (s, 3 H), 3.96 (s, 3 H), 3.97 (s, 3 H), 3.98 (s, 3 H), 3.99 (s, 3 H), 4.19 (s, 3 H), 4.22 (s, 3 H), 5.87 (s, 1 H), 6.04 (s, 1 H), 7.27 (d, 1 H, *J = *8.8 Hz), 7.32 (d, 1 H, *J = *8.8 Hz), 7.61 (d, 1 H, *J = *8.8 Hz), 7.82 (d, 1 H, *J = *8.8 Hz); ^13^C NMR (CDCl_3_): δ 56.7, 57.1, 60.7, 61.1, 61.2, 61.3, 101.3, 106.5, 119.5, 119.7, 120.4, 121.5, 122.1, 124.2, 130.9, 131.1, 131.8, 132.0, 132.4, 132.5, 132.7, 133.8, 146.9, 147.0, 150.5, 150.8, 154.0, 154.3, 163.0, 169.2, 182.3, 184.4, 185.0, 186.7; HRESIMS *m/z* 677.1639 (calculated for C_36_H_30_O_12_Na, 677.1635).

#### 5-Hydroxy-3,6,7-trimethoxy-1,4-phenanthrenequinone; calanquinone A (6a)

Compound **5a** (115.02 mg, 0.35 mmol) was dissolved in 5 mL CH_2_Cl_2_ and iodotrimethylsilane (112.23 mg, 0.56 mmol) was added to the solution in portions. The mixture was stirred at 60°C overnight (TLC monitoring) and then MeOH was added to quench the reaction. The solvent was evaporated and the residue extracted with Et_2_O/H_2_O. The Et_2_O layer was dried over Na_2_SO_4_. The mixture was chromatographed on silica gel and eluted with EtOAc/*n*-hexane (1∶2) to yield **6a** (15.72 mg, 14.28%). Black solid; mp 187°C; ^1^H NMR (CDCl_3_): δ 3.96 (s, 3 H), 4.01 (s, 3 H), 4.02 (s, 3 H), 6.14 (s, 1 H), 6.85 (s, 1 H), 8.03 (d, 1 H, *J = *8.4 Hz), 8.08 (d, 1 H, *J = *8.4 Hz), 10.73 (s, OH); ^13^C NMR (CDCl_3_): δ 55.9, 56.9, 60.8, 101.2, 107.2, 118.5, 121.7, 128.1, 132.8, 134.8, 136.9, 140.1, 148.1, 155.0, 161.5, 184.4, 186.0; HRESIMS *m/z* 313.0648 (calculated for C_17_H_14_O_6_ -H, 313.0712).

#### 5-Hydroxy-3,7-dimethoxy-1,4-phenanthrenequinone; denbinobin (6b)

Compound **5b** (67.56 mg, 0.23 mmol) and iodotrimethylsilane were used with the method described for **6a**. The crude product was chromatographed on silica gel and eluted with CH_2_Cl_2_ to yield **6b** (6.0 mg, 10%). Black solid; mp 215°C [21]; ^1^H NMR (CDCl_3_): δ 3.93 (s, 3 H), 3.96 (s, 3 H), 6.15 (s, 1 H), 6.82 (d, 1 H, *J = *2.8 Hz), 6.93 (d, 1 H, *J = *2.8 Hz), 8.06 (d, 1 H, *J = *8.8 Hz), 8.12 (d, 1 H, *J = *8.8 Hz), 11.00 (s, OH); ^13^C NMR (CDCl_3_): δ 55.5, 56.9, 101.8, 107.3, 108.7, 117.2, 122.6, 128.6, 132.4, 137.4, 139.9, 156.4, 160.8, 161.2, 184.4, 186.5; HRESIMS *m/z* 307.0584 (calculated for C_16_H_12_O_5_ Na, 307.0582).

#### 5-Acetoxy-3,6,7-trimethoxy-1,4-phenanthrenequinone (7a) and 5-acetoxy-3,7-dimethoxy-1,4-phenanthrenequinone (7b)

Compounds **6a** (3.29 mg, 0.01 mmol) and **6b** (2.03 mg, 0.007 mmol) were each dissolved in pyridine and excess Ac_2_O was added to the two solutions. The mixture was stirred at RT overnight and the pyridine was evaporated. The crude material was chromatographed on silica gel and eluted with EtOAc/*n*-hexane (1∶2) to afford **7a** (4.65 mg, 124.66%) and **7b** (2.80 mg, 120.10%), respectively.

Data for **7a**: Yellow green solid; mp 196°C; ^1^H NMR (CDCl_3_): δ 2.36 (s, 3 H), 3.93 (s, 3 H), 3.99 (s, 3 H), 4.03 (s, 3 H), 6.05 (s, 1 H), 7.15 (s, 1 H), 7.97 (d, 1 H, *J* = 8.8 Hz), 8.07 (d, 1 H, *J* = 8.8 Hz); ^13^C NMR (CDCl_3_): δ 20.8, 56.0, 56.6, 60.8, 106.0, 106.4, 119.4, 121.6, 130.2, 132.1, 133.1, 134.1, 139.7, 144.2, 154.9, 162.7, 168.4, 181.8, 184.4; HRESIMS *m/z* 379.0791 (calculated for C_19_H_16_O_7_Na, 379.0794).

Data for **7b**: Yellow solid; mp 201°C; ^1^H NMR (CDCl_3_): δ 2.32 (s, 3 H), 3.93 (s, 3 H), 3.96 (s, 3 H), 6.06 (s, 1 H), 7.10 (d, 1 H, *J* = 2.4 Hz), 7.15 (d, 1 H, *J* = 2.4 Hz), 8.00 (d, 1 H, *J* = 8.4 Hz), 8.11 (d, 1 H, *J* = 8.4 Hz); ^13^C NMR (CDCl_3_): δ 21.1, 55.8, 56.6, 105.3, 106.5, 116.3, 118.2, 122.6, 131.0, 131.6, 133.3, 138.8, 148.0, 159.6, 162.5, 168.8, 181.9, 184.4; HRESIMS *m/z* 349.0686 (calculated for C_18_H_14_O_6_Na, 349.0688).

#### 5,6-Dihydroxy-1,4,7-trimethoxy-phenanthrene (8a) and 5-hydroxy-1,4,6,7-tetramethoxy-phenanthrene (9a)

Compound **3a** (109.39 mg, 0.33 mmol) was dissolved in benzene, and AlCl_3_ (987 mg, 7.40 mmol) was added to the solution in portions. The mixture was stirred at 70°C overnight (TLC monitoring) and poured into 50 mL of ice-water along with 5 mL concentrated hydrochloric acid [14]. The suspension was extracted with CH_2_Cl_2_ and dried over Na_2_SO_4_. The crude material was chromatographed on silica gel and eluted with EtOAc/*n*-hexane (1∶2) to yield **8a** (44.49 mg, 44.47%) and **9a** (9.33 mg, 8.91%).

Data for **8a**: Pale brownish solid; mp 116°C; ^1^H NMR (CDCl_3_): δ 3.94 (s, 3 H), 4.01 (s, 3 H), 4.06 (s, 3 H), 6.33 (s, OH), 6.95 (d, 1 H, *J* = 8.4 Hz), 6.98 (s, 1 H), 7.20 (d, 1 H, *J* = 8.4 Hz), 7.60 (d, 1 H, *J* = 9.6 Hz), 8.03 (d, 1 H, *J* = 9.6 Hz), 10.77 (s, OH); ^13^C NMR (CDCl_3_): δ 56.0, 56.1, 60.7, 101.6, 105.3, 113.2, 113.5, 117.8, 121.0, 124.7, 127.6, 127.7, 135.3, 140.6, 147.7, 147.8, 152.0; HRESIMS *m/z* 323.0894 (calculated for C_17_H_17_O_5_Na, 323.0895).

Data for **9a**: Pale yellowish solid; mp 124°C; ^1^H NMR (CDCl_3_): δ 3.94 (s, 3 H), 3.99 (s, 3 H), 4.01 (s, 3 H), 4.03 (s, 3 H), 6.93 (s, 1 H), 6.96 (d, 1 H, *J* = 8.4 Hz), 7.21 (d, 1 H, *J* = 8.4 Hz), 7.59 (d, 1 H, *J* = 9 Hz), 8.10 (d, 1 H, *J* = 9 Hz), 10.52 (s, OH); ^13^C NMR (CDCl_3_): δ 55.8, 56.2, 60.3, 60.8, 101.1, 105.4, 113.7, 114.2, 119.4, 121.6, 124.5, 127.5, 131.1, 137.9, 147.7, 149.0, 151.9, 153.2; HRESIMS *m/z* 337.1054 (calculated for C_18_H_18_O_5_Na, 337.1052).

#### 4-Hydroxy-1,5,7-trimethoxy-phenanthrene (8b) and 5-hydroxy-1,4,7-trimethoxy-phenanthrene (9b)

Compound **3b** (91.65 mg, 0.32 mmol), AlCl_3_ (509.60 mg, 3.82 mmol), and benzene were used with the method described for **8a** and **9a**. The crude was chromatographed on silica gel and eluted with EtOAc/*n*-hexane (1∶7) to yield **8b** (20.00 mg, 22.77%) and **9b** (22.00 mg, 24.9%).

Data for **8b**: Pale brownish solid; mp 71°C; ^1^H NMR (CDCl_3_): δ 3.97 (s, 3 H), 3.99 (s, 3 H), 4.06 (s, 3 H), 6.85 (d, 1 H, *J* = 2.4 Hz), 7.00 (d, 1 H, *J* = 8.8 Hz), 7.01 (d, 1 H, *J* = 2.4 Hz), 7.16 (d, 1 H, *J* = 8.8 Hz), 7.55 (d, 1 H, *J* = 8.8 Hz), 8.21 (d, 1 H, *J* = 8.8 Hz), 9.12 (s, OH); ^13^C NMR (CDCl_3_): δ 55.6, 56.4, 58.2, 101.6, 103.4, 107.6, 114.3, 115.6, 119.8, 122.3, 124.1, 125.7, 136.2, 147.5, 149.1, 155.5, 158.3; HRESIMS *m/z* 307.0948 (calculated for C_17_H_16_O_4_Na, 307.0946).

Data for **9b**: Pale brownish solid; mp 70°C; ^1^H NMR (CDCl_3_): δ 3.92 (s, 3 H), 3.94 (s, 3 H), 4.00 (s, 3 H), 6.89 (d, 1 H, *J* = 2.8 Hz), 6.92 (d, 1 H, *J* = 2.8 Hz), 6.94 (d, 1 H, *J* = 8.8 Hz), 7.20 (d, 1 H, *J* = 8.8 Hz), 7.61 (d, 1 H, *J* = 8.8 Hz), 8.13 (d, 1 H, *J* = 8.8Hz), 10.38 (s, OH); ^13^C NMR (CDCl_3_): δ 55.3, 56.1, 60.4, 102.5, 104.7, 105.4, 113.1, 114.2, 120.4, 121.8, 124.3, 127.8, 136.4, 147.6, 151.9, 156.4, 159.3; HRESIMS *m/z* 307.0947 (calculated for C_17_H_16_O_4_Na, 307.0946).

## Supporting Information

Figure S1 Pharmacophore of run 19 maps with 6a.
(TIF)

Table S1 The different parameters employed in each run.
(DOC)

Table S2 The pharmacophore results of the best hypothesis in each run.
(DOC)

Table S3 Experimental and predictive values of the compounds in the pharmacophore model.
(DOC)

Table S4 The values of molecular properties used to describe the effects of molecular solubility and transportation.
(DOC)

Text S1 Pharmacophore built with Catalyst HypoGen.
(DOC)
