# Supplementary figures and images for: Synthesis and Biological Evaluation of Phenanthrenes as Cytotoxic Agents with Pharmacophore Modeling and ChemGPS-NP Prediction as Topo II Inhibitors
Source: PLoS One. 2012 May 29;7(5):e37897. doi: 10.1371/journal.pone.0037897 (PMC3362575; doi:10.1371/journal.pone.0037897)

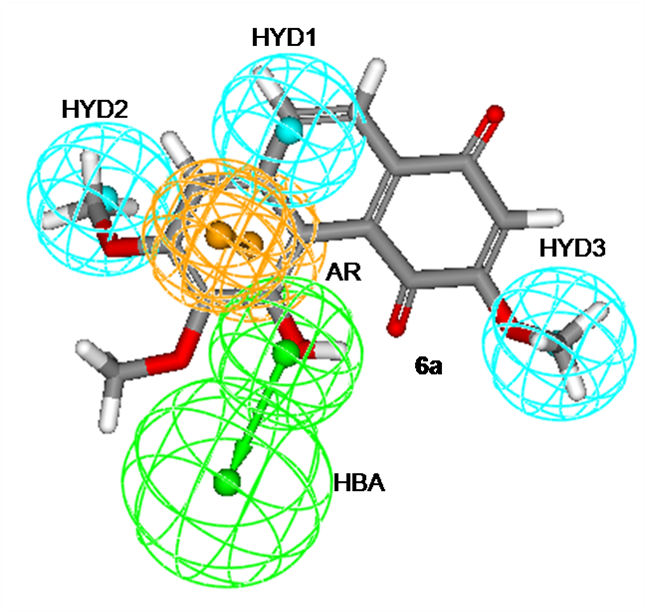

Supplement: Figure S1 — Pharmacophore of run 19 maps with 6a. (TIF) [file pone.0037897.s001.tif]
