# Supplementary material for: Synthesis and Biological Evaluation of Phenanthrenes as Cytotoxic Agents with Pharmacophore Modeling and ChemGPS-NP Prediction as Topo II Inhibitors
Source: PLoS One. 2012 May 29;7(5):e37897. doi: 10.1371/journal.pone.0037897 (PMC3362575; doi:10.1371/journal.pone.0037897)
Supplement: Table S1 — The different parameters employed in each run. (DOC) [file pone.0037897.s002.doc]

**Table S1 The different parameters employed in each run.**

| Run | Feature types and ranges*^a^* | Uncertainty | Advanced parameter*^b^* | | |  |
| --- | --- | --- | --- | --- | --- | --- |
| 1 | HBA (0-5), HYD (0-5), AR (0-5), HBD (0-5) | 3 | None |  |  |  |
| 2 | HBA (0-5), HYD (0-5), AR (0-5), HBD (0-5) | 3 | Variable tolerance |  |  |  |
| 3 | HBA (0-5), HYD (0-5), AR (0-5), HBD (0-5) | 3 | Variable weight |  |  |  |
| 4 | HBA (0-5), HYD (0-5), AR (0-5) | 3 | None |  |  |  |
| 5 | HBA (0-5), HYD (0-5), AR (0-5) | 3 | Variable tolerance |  |  |  |
| 6 | HBA (0-5), HYD (0-5), AR (0-5) | 3 | Variable weight |  |  |  |
| 7 | HBA (0-5), HYD (0-5) | 3 | None |  |  |  |
| 8 | HBA (0-5), HYD (0-5) | 3 | Variable tolerance |  |  |  |
| 9 | HBA (0-5), HYD (0-5) | 3 | Variable weight |  |  |  |
| 10 | HBA (0-5), AR (0-5) | 3 | None |  |  |  |
| 11 | HBA (0-5), AR (0-5) | 3 | Variable tolerance |  |  |  |
| 12 | HBA (0-5), AR (0-5) | 3 | Variable weight |  |  |  |
| 13 | HBA (0-5) | 3 | None |  |  |  |
| 14 | HBA (0-5) | 3 | Variable tolerance |  |  |  |
| 15 | HBA (0-5) | 3 | Variable weight |  |  |  |
| 16 | HBA (0-5), HYD (0-5), AR (0-5), HBD (0-5) | 2 | None |  |  |  |
| 17 | HBA (0-5), HYD (0-5), AR (0-5), HBD (0-5) | 2 | Variable tolerance |  |  |  |
| 18 | HBA (0-5), HYD (0-5), AR (0-5), HBD (0-5) | 2 | Variable weight |  |  |  |
| 19 | HBA (0-5), HYD (0-5), AR (0-5) | 2 | None |  |  |  |
| 20 | HBA (0-5), HYD (0-5), AR (0-5) | 2 | Variable tolerance |  |  |  |
| 21 | HBA (0-5), HYD (0-5), AR (0-5) | 2 | Variable weight |  |  |  |
| 22 | HBA (0-5), HYD (0-5) | 2 | None |  |  |  |
| 23 | HBA (0-5), HYD (0-5) | 2 | Variable tolerance |  |  |  |
| 24 | HBA (0-5), HYD (0-5) | 2 | Variable weight |  |  |  |
| 25 | HBA (0-5), AR (0-5) | 2 | None |  |  |  |
| 26 | HBA (0-5), AR (0-5) | 2 | Variable tolerance |  |  |  |
| 27 | HBA (0-5), AR (0-5) | 2 | Variable weight |  |  |  |
| 28 | HBA (0-5) | 2 | None |  |  |  |
| 29 | HBA (0-5) | 2 | Variable tolerance |  |  |  |
| 30 | HBA (0-5) | 2 | Variable weight |  |  |  |

*^a^* HBD = hydrogen bond donor, HBA = hydrogen bond acceptor, HYD = hydrophobic, AR =aromatic ring. *^b^* Specifies whether advanced parameters, variable tolerance and variable weight were employed in the modeling run.
