# Supplementary material for: Synthesis and Biological Evaluation of Phenanthrenes as Cytotoxic Agents with Pharmacophore Modeling and ChemGPS-NP Prediction as Topo II Inhibitors
Source: PLoS One. 2012 May 29;7(5):e37897. doi: 10.1371/journal.pone.0037897 (PMC3362575; doi:10.1371/journal.pone.0037897)
Supplement: Table S2 — The pharmacophore results of the best hypothesis in each run. (DOC) [file pone.0037897.s003.doc]

**Table S2** The pharmacophore results of the best hypothesis in each run.

| Run | feature | Cost values | | | | RMS | R*^c^* | Tol.*^d^* | Null*^e^* ∆cost*^f^* | | F*^g^* (%) | |
| --- | --- | --- | --- | --- | --- | --- | --- | --- | --- | --- | --- | --- |
|  |  | Total | Error | W*^a^* | Config.*^b^* |  |  |  |  |  |  |  |
| 1 | HBA×2, HYD×2 | 119.191 | 102.618 | 1.176 | 15.397 | 0.592 | 0.901 | 0 | 124.452 | 5.261 | 95 |  |
| 2 | HBA×2, HYD×2 | 133.625 | 103.498 | 1.255 | 27.007 | 0.641 | 0.883 | 1.865 | 124.452 | 9.173 | 95 |  |
| 3 | HBA×2, HYD, AR | 130.905 | 102.734 | 1.164 | 27.007 | 0.598 | 0.899 | 0 | 124.452 | 6.453 | 95 |  |
| 4 | HBA×2, HYD×2 | 119.221 | 102.713 | 1.162 | 15.346 | 0.597 | 0.899 | 0 | 124.452 | 5.231 | 95 |  |
| 5 | HBA×2, HYD×2 | 131.849 | 102.314 | 1.157 | 26.955 | 0.574 | 0.907 | 1.423 | 124.452 | 7.397 | 95 |  |
| 6 | HBA×2, HYD×2 | 130.503 | 102.068 | 1.480 | 26.955 | 0.559 | 0.918 | 0 | 124.452 | 6.051 | 95 |  |
| 7 | HBA×2, HYD×2 | 123.297 | 107.927 | 1.126 | 14.244 | 0.846 | 0.784 | 0 | 124.452 | 1.155 | 95 |  |
| 8 | HBA×2, HYD×2 | 133.091 | 104.395 | 1.128 | 25.854 | 0.687 | 0.863 | 1.715 | 124.452 | 8.639 | 95 |  |
| 9 | HBA×2, HYD×2 | 129.806 | 102.145 | 1.807 | 25.854 | 0.563 | 0.920 | 0 | 124.452 | 5.354 | 95 |  |
| 10 | HBA×3 | 115.243 | 101.613 | 1.733 | 11.897 | 0.530 | 0.926 | 0 | 124.452 | 9.209 | 95 |  |
| 11 | HBA×3 | 126.496 | 100.370 | 1.460 | 23.506 | 0.441 | 0.948 | 1.159 | 124.452 | 2.043 | 95 |  |
| 12 | HBA×3 | 127.089 | 102.083 | 1.500 | 23.506 | 0.559 | 0.919 | 0 | 124.452 | 2.637 | 95 |  |
| 13 | HBA×3 | 114.177 | 102.239 | 1.607 | 10.331 | 0.569 | 0.912 | 0 | 124.452 | 10.275 | 95 |  |
| 14 | HBA×3 | 126.184 | 101.008 | 1.865 | 21.941 | 0.489 | 0.939 | 1.370 | 124.452 | 1.732 | 95 |  |
| 15 | HBA×3 | 125.004 | 101.290 | 1.773 | 21.941 | 0.508 | 0.945 | 0 | 124.452 | 0.552 | 95 |  |
| 16 | HBA×2, HYD, AR | 114.364 | 97.822 | 1.138 | 15.403 | 0.970 | 0.893 | 0 | 151.783 | 37.419 | 95 |  |
| 17 | HBA×3, HYD | 121.462 | 91.628 | 1.178 | 27.013 | 0.716 | 0.943 | 1.643 | 151.783 | 30.321 | 95 |  |
| 18 | HBA×2, HYD×2 | 121.927 | 92.072 | 2.842 | 27.012 | 0.737 | 0.942 | 0 | 151.783 | 29.856 | 95 |  |
| 19 | HBA, HYD×3, AR | 109.525 | 92.720 | 1.459 | 15.346 | 0.767 | 0.935 | 0 | 151.783 | 42.258 | 95 |  |
| 20 | HBA×3, HYD | 120.874 | 90.513 | 2.105 | 26.956 | 0.660 | 0.953 | 1.300 | 151.783 | 30.909 | 95 |  |
| 21 | HBA×2, HYD, AR | 123.159 | 95.063 | 1.141 | 26.956 | 0.866 | 0.916 | 0 | 151.783 | 28.624 | 95 |  |
| **22** | **HBA×3, HYD** | **109.366** | **93.243** | **1.879** | **14.244** | **0.790** | **0.931** | **0** | **151.783** | **42.417** | **95** |  |
| 23 | HBA×3, HYD | 120.747 | 91.607 | 1.350 | 25.854 | 0.715 | 0.944 | 1.936 | 151.783 | 31.036 | 95 |  |
| 24 | HBA×2, HYD×2 | 123.389 | 95.678 | 1.857 | 25.854 | 0.890 | 0.914 | 0 | 151.783 | 28.394 | 95 |  |
| 25 | HBA×3 | 108.224 | 93.548 | 2.780 | 11.897 | 0.803 | 0.931 | 0 | 151.783 | 43.559 | 95 |  |
| 26 | HBA×3 | 115.555 | 88.654 | 2.235 | 23.506 | 0.555 | 0.968 | 1.159 | 151.783 | 36.228 | 95 |  |
| 27 | HBA×3 | 120.094 | 92.936 | 3.652 | 23.506 | 0.777 | 0.938 | 0 | 151.783 | 31.689 | 95 |  |
| 28 | HBA×3 | 108.314 | 95.499 | 2.483 | 10.331 | 0.883 | 0.914 | 0 | 151.783 | 43.469 | 95 |  |
| 29 | HBA×3 | 114.259 | 88.882 | 2.233 | 21.941 | 0.569 | 0.966 | 1.204 | 151.783 | 37.524 | 95 |  |
| 30 | HBA×3 | 116.169 | 90.807 | 3.421 | 21.941 | 0.676 | 0.955 | 0 | 151.783 | 35.614 | 95 |  |

*^a^* Variable feature weight. *^b^* Configuration cost. *^c^* The correlation coefficients between the estimated and experimental bioactivities. *^d^* Variable feature tolerance. *^e^* Null cost. *^f^* The difference between the total cost and the null cost. *^g^* Fischer′s randomization test was calculated by Cat-Scramble methods at a 95% confidence level.
