# Supplementary material for: Synthesis and Biological Evaluation of Phenanthrenes as Cytotoxic Agents with Pharmacophore Modeling and ChemGPS-NP Prediction as Topo II Inhibitors
Source: PLoS One. 2012 May 29;7(5):e37897. doi: 10.1371/journal.pone.0037897 (PMC3362575; doi:10.1371/journal.pone.0037897)
Supplement: Table S3 — Experimental and predictive values of the compounds in the pharmacophore model. (DOC) [file pone.0037897.s004.doc]

**Table S3 Experimental and predictive values of the compounds in the pharmacophore model study.**

|  | IC_50_ (μg/mL) (MCF-7) | |  |  | Scale*^c^* | |  |
| --- | --- | --- | --- | --- | --- | --- | --- |
| Compd | Exptl. | Estimated | Error*^a^* | Fit value*^b^* | Exptl. | Estimated | AlogP*^d^* |
| **CA-2** | 11.90 | 7.58 | - 1.6 | 6.87 | ++ | ++ | 2.08 |
| **CA-3** | 13.25 | 8.26 | - 1.6 | 6.83 | ++ | ++ | 2.45 |
| **CA-4** | 14.30 | 9.94 | - 1.4 | 6.75 | ++ | ++ | 3.19 |
| **CA-5** | 12.70 | 10.82 | - 1.2 | 6.72 | ++ | ++ | 3.19 |
| **CA-6** | 20.00 | 12.01 | - 1.7 | 6.67 | + | ++ | 3.42 |
| **CA-7** | 14.62 | 9.83 | - 1.5 | 6.76 | ++ | ++ | 3.43 |
| **CA-8** | 20.00 | 11.68 | - 1.7 | 6.68 | + | ++ | 3.13 |
| **CA-9** | 10.25 | 18.98 | +1.9 | 6.47 | ++ | ++ | 3.43 |
| **CA-10** | 6.77 | 20.63 | +3.0 | 6.44 | ++ | + | 3.45 |
| **CA-11** | 4.84 | 10.70 | +2.2 | 6.72 | ++ | ++ | 6.59 |
| **3a** | 20.00 | 25.43 | +1.3 | 6.35 | + | + | 3.56 |
| **3b** | 20.00 | 36.86 | +1.8 | 6.18 | + | + | 3.58 |
| **4a** | 13.19 | 10.26 | - 1.3 | 6.74 | ++ | ++ | 2.62 |
| **4b** | 15.63 | 23.36 | +1.5 | 6.38 | ++ | + | 2.63 |
| **4c** | 4.71 | 11.34 | +2.4 | 6.70 | ++ | ++ | 4.92 |
| **5a** | 14.29 | 8.59 | - 1.7 | 6.82 | ++ | ++ | 2.30 |
| **5b** | 14.40 | 8.10 | - 1.8 | 6.84 | ++ | ++ | 2.32 |
| **5c** | 11.10 | 7.40 | - 1.5 | 6.88 | ++ | ++ | 2.12 |
| **5d** | 13.78 | 6.94 | - 2.0 | 6.91 | ++ | ++ | 4.30 |
| **5e** | 9.40 | 6.09 | - 1.5 | 6.97 | ++ | ++ | 4.09 |
| **5f** | 14.77 | 7.38 | - 2.0 | 6.88 | ++ | ++ | 4.29 |
| **6a** (**CA-1**) | 0.09 | 0.15 | +1.7 | 8.57 | +++ | +++ | 2.08 |
| **6b** | 0.26 | 0.63 | +2.4 | 7.95 | +++ | +++ | 2.10 |
| **7a** | 0.16 | 0.25 | +1.5 | 8.36 | +++ | +++ | 2.09 |
| **7b** | 0.53 | 0.56 | +1.1 | 8.00 | +++ | +++ | 2.11 |
| **8a** | 17.01 | 16.72 | 1.0 | 6.53 | ++ | ++ | 3.11 |
| **8b** | 19.31 | 28.82 | +1.5 | 6.29 | ++ | + | 3.36 |
| **9a** | 20.00 | 17.31 | - 1.2 | 6.51 | + | ++ | 3.34 |
| **9b** | 20.00 | 12.63 | - 1.6 | 6.65 | + | ++ | 3.36 |

*^a^* + and - indicate that the estimated IC_50_ values are either higher or lower than the experimental IC_50_, respectively; a value of 1 indicates that the estimated IC_50_ is equal to the experimental IC_50_. *^b^* Fit values indicate how well the features in the pharmacophore map the chemical features in the molecule. *^c^* These compounds are classified by their activity as highly active (IC_50_ < 1.0 μg/mL, +++), moderately active (1.0 μg/mL < IC_50_ < 20.0 μg/mL, ++), or inactive (IC_50_ > 20.0 μg/mL, +). *^d^* AlogP means the logarithm of the octanol-water partition coefficient using Ghose and Crippen's method.
