# Supplementary material for: Synthesis and Biological Evaluation of Phenanthrenes as Cytotoxic Agents with Pharmacophore Modeling and ChemGPS-NP Prediction as Topo II Inhibitors
Source: PLoS One. 2012 May 29;7(5):e37897. doi: 10.1371/journal.pone.0037897 (PMC3362575; doi:10.1371/journal.pone.0037897)
Supplement: Table S4 — The values of molecular properties used to describe the effects of molecular solubility and transportation. (DOC) [file pone.0037897.s005.doc]

**Table S4** The values of molecular properties used to describe the effects of molecular solubility and transportation

| Compd | pIC_50_*^a^* | ALogP*^b^* | Molecular Solubility*^c^* | Molecular PSA*^d^* |
| --- | --- | --- | --- | --- |
| **CA-2** | -1.08 | 2.08 | -3.25 | 3.00 |
| **CA-3** | -1.12 | 2.45 | -2.76 | 2.00 |
| **CA-4** | -1.16 | 3.19 | -2.62 | 2.00 |
| **CA-5** | -1.10 | 3.19 | -2.62 | 2.00 |
| **CA-6** | -1.30 | 3.42 | -3.30 | 3.00 |
| **CA-7** | -1.16 | 3.43 | -3.39 | 2.00 |
| **CA-8** | -1.30 | 3.13 | -3.68 | 2.00 |
| **CA-9** | -1.01 | 3.43 | -3.52 | 2.00 |
| **CA-10** | -0.83 | 3.45 | -3.51 | 1.00 |
| **CA-11** | -0.68 | 6.59 | -6.98 | 3.00 |
| **3a** | -1.30 | 3.56 | -5.20 | 5.00 |
| **3b** | -1.30 | 3.58 | -5.24 | 4.00 |
| **4a** | -1.12 | 2.62 | -3.92 | 3.00 |
| **4b** | -1.19 | 2.63 | -3.94 | 2.00 |
| **4c** | -0.67 | 4.92 | -7.57 | 7.00 |
| **5a** | -1.16 | 2.30 | -3.90 | 4.00 |
| **5b** | -1.16 | 2.32 | -3.93 | 3.00 |
| **5c** | -1.05 | 2.12 | -3.61 | 4.00 |
| **5d** | -1.14 | 4.30 | -7.44 | 9.00 |
| **5e** | -0.97 | 4.09 | -6.94 | 10.00 |
| **5f** | -1.17 | 4.29 | -7.56 | 9.00 |
| **6a (CA-1)** | 1.05 | 2.08 | -3.22 | 3.00 |
| **6b** | 0.59 | 2.10 | -3.26 | 2.00 |
| **7a** | 0.80 | 2.09 | -4.03 | 5.00 |
| **7b** | 0.28 | 2.11 | -4.08 | 4.00 |
| **8a** | -1.23 | 3.11 | -3.68 | 3.00 |
| **8b** | -1.29 | 3.36 | -4.44 | 3.00 |
| **9a** | -1.30 | 3.34 | -4.38 | 4.00 |
| **9b** | -1.30 | 3.36 | -4.44 | 3.00 |

*^a^* pIC_50_ is the negative logarithm of the IC_50_ value. *^b^* AlogP is the logarithm of the octanol-water partition coefficient. *^c^* Molecular Solubility is expressed as logS, where S is water solubility in mol/L. *^d^* Molecular PSA is the polar surface area for each molecule, standing for transportation effect.
