# Supplementary material for: Synthesis and Biological Evaluation of Phenanthrenes as Cytotoxic Agents with Pharmacophore Modeling and ChemGPS-NP Prediction as Topo II Inhibitors
Source: PLoS One. 2012 May 29;7(5):e37897. doi: 10.1371/journal.pone.0037897 (PMC3362575; doi:10.1371/journal.pone.0037897)
Supplement: Text S1 — Pharmacophore built with Catalyst HypoGen. (DOC) [file pone.0037897.s006.doc]

**SUPPORTING INFORMATION for**

**Synthesis and Biological Evaluation of Phenanthrenes as Cytotoxic Agents with Pharmacophore Modeling and ChemGPS-NP Prediction as Topo II Inhibitors**

**Chia-Lin Lee^1,2,†^, Ying-Ting Lin^3,†^, Fang-Rong Chang^4,5^,*, Guan-Yu Chen^2^, Anders Backlund^6^, Juan-Chang Yang^4^, Shu-Li Chen^4^, Yang-Chang Wu^1,2,4,7^,***

**1** School of Chinese Medicine, China Medical University, Taichung, Taiwan, **2** Natural Medicinal Products Research Center, China Medical University Hospital, Taichung, Taiwan, **3** Department of Biotechnology, Kaohsiung Medical University, Kaohsiung, Taiwan, **4** Graduate Institute of Natural Products, Kaohsiung Medical University, Kaohsiung, Taiwan, **5** Cancer Center, Kaohsiung Medical University Hospital, Kaohsiung, Taiwan, **6** Division of Pharmacognosy, Department of Medicinal Chemistry, BMC, Uppsala University, Uppsala, Sweden, **7** Center for Molecular Medicine, China Medical University Hospital, Taichung, Taiwan

* E-mail: [yachwu@mail.cmu.edu.tw](mailto:yachwu@mail.cmu.edu.tw) (YCW); [aaronfrc@kmu.edu.tw](mailto:aaronfrc@kmu.edu.tw) (FRC)

^†^ These authors contributed equally to this work.

**Contents**

Figure S1 Pharmacophore of run 19 maps with **6a**

Table S1 The different parameters employed in each run

Table S2 The pharmacophore results of the best hypothesis in each run

Table S3 Experimental and predictive values of the compounds in the pharmacophore model

Table S4 The values of molecular properties used to describe the effects of molecular solubility and transportation

References

**Pharmacophore built with Catalyst HypoGen**

The best chosen pharmacophore out of 30 HypoGen runs containing three hydrogen-bond acceptors and one hydrophobic feature, run 22 (*r* = 0.931), was built based on 29 compounds and their MCF-7 cytotoxic activity (IC_50_ values). The best run was chosen from 30 runs with various parameters that were conducted in advance. Table S1 shows the 30 runs with some significant parameter adjustments, type selection [hydrogen-bond acceptor (HBA), hydrogen-bond donor (HBD), hydrophobic (HYD), and aromatic ring (AR)], the size of feature number (0-5), uncertainty value (referring to the range of compound activity) (2 or 3), variable tolerance and weight (improving the correlation with activity and the searching result).

Table S2 presents hypothesis results from 30 HypoGen runs that used different settings. The table includes feature types, cost data [total cost, error cost, weight cost, configuration cost, tolerance cost, null cost and the difference between the total cost and the null cost (∆cost)], root mean square (RMS), and the correlation coefficients (R) between the estimated and experimental bioactivities. Generally, the highest ∆cost value gets the best significant hypothesis. Fischer′s randomization test (F) was calculated by Cat-Scramble methods in catalyst at a 95% confidence level to confirm whether there was a strong correlation and cross validation between the structures and the logarithm of cytotoxic activities.

Run 22, highlighted in Table S2, was the best pharmacophore model in 30 runs according to the following considerations:

Features of each result were selected after a HypoGen run shown in Table S2. Notice that HBD didn′t appear in all models. Both HBA and HYD clearly emerged after running HypoGen as they were selected for use. On the other hand, our compounds not only have phenanthrene, phenanthrenequinone skeletons and the methyl groups of methoxyl substituents that can affect the processing of the hydrophobic characteristics but also contain the oxygen atoms of carbonyl, methoxyl and/or hydroxyl groups as the features of HBA. Thus, HBA and HYD are considered to be necessary and potent features that contribute to the biological activity in this series of compounds.

In cost analysis, ∆cost ranged from 40 to 60, which represents a true correlation of the pharmacophore hypothesis with 75-90% high probabilities. The true correlation represents a < 50% probability when it is less than 40. Generally, the configuration cost should be smaller than 17 in a standard HypoGen model.

To analyze “cost” data in Table S3, four models of runs 19 (configuration cost = 15.346 < 17, ∆cost= 42.258 > 40), 22 (configuration cost = 14.244 < 17, ∆cost= 42.417 > 40), 25 (configuration cost = 11.897 < 17, ∆cost= 43.559 > 40) and 28 (configuration cost = 10.331 < 17, ∆cost= 43.469 > 40) qualified. Run 19 (Figure S1) had the highest correlation coefficient (*r* = 0.935) between the estimated and experimental bioactivities, but it possesses one HBA, three HYD and one AR feature. The three HYDs and one AR, for a total of four features, belong to the hydrophobic nature, and AR can basically be substituted by HYD. Moreover, HYD1, HYD2 and AR are somehow mutually collided in a single pharmacophore and most importantly, run 19 has no feature that addresses the quinone motif; however, quinone motif was considered essential for high cytotoxicity. Therefore, run 19 is a subordinate model. Runs 25 and 28 were not suitable models due to their lack of an HYD feature needed for losing the hydrophobic anchor representing the phenanthrene skeleton, i.e., the three hydrophobic rings, for the series.

Notice that run 26 possesses the highest correlation coefficient (*r* = 0.968) among the 30 runs (Table S2), but the difference between total cost and null cost for this run was less than 40 and the configuration cost was larger than 17.

Taken together, run 22, which possesses three HBA and one HYD feature, was considered by cost analysis and chosen as the best pharmacophore hypothesis.

To test the run 22 model, the IC_50_ values spanning from 0.09 to 20 μg/mL were classified as highly active (IC_50_ < 1.0 μg/mL, +++), moderately active (1.0 μg/mL < IC_50_ < 20.0 μg/mL, ++), and inactive (IC_50_ > 20.0 μg/mL, +). As shown in Table S3, all compounds were correctly predicted except **CA-6**, **CA-8**, **CA-10**, **4b**, **8b**, **9a** and **9b**. Three ++ (**CA-10**, **4b**, **8b**) and four + (**CA-6**, **CA-8**, **9a**, **9b**) compounds were incorrectly predicted in the + and ++ categories, respectively. Additionally, all +++ compounds were correctly predicted in the +++ categories. The accuracy and correlation coefficient (*r*) between the estimated and experimental IC_50_ values reached 75.86% and 0.931, respectively, indicating that the model of run 22 showed a significant correlation between the structure and biological property.

As shown in Table S4, the molecular properties, including AlogP (the logarithm of the octanol-water partition coefficient, used to describe the hydrophobicity of the molecule),[^1^](#_ENREF_1) molecular solubility (logS, taking logarithm of the water solubility in mol/L)[^2^](#_ENREF_2) and molecular PSA (the polar surface area of each molecule stands for transportation for a drug),[^3^](#_ENREF_3) were chosen to describe the molecular solubility and transportation effect in the bioassay. The molecular properties and their correlation coefficients to activities were calculated by the software *Discovery Studio version 2.1*. and MS Excel, respectively. To the results in Table S4, the properties of the molecular solubility and transportation effect are not the major factors for the efficacy of compounds due to the low correlation coefficients with activity [*r* = -0.31 (pIC_50_-AlogP), *r* = 0.10 (pIC_50_-molecular solubility), *r* = 0.17 (pIC_50_-molecular PSA)]. From our modeling, the molecular structure can be thus the major factor.

**References**

1. Ghose AK, Crippen GM (1987) J Chem Inf Comput Sci 27: 21–35.
2. Tetko IV, Tanchuk VY, Kasheva TN, Villa AE (2001) J Chem Inf Comput Sci 41: 1488–1493.
3. Ertl P, Rohde B, Selzer P (2000) J. Med. Chem. 43: 3714–3717.
